# Co-Transcriptomes of Initial Interactions *In Vitro* between *Streptococcus Pneumoniae* and Human Pleural Mesothelial Cells

**DOI:** 10.1371/journal.pone.0142773

**Published:** 2015-11-13

**Authors:** Claire J. Heath, Maria del Mar Cendra, Alastair Watson, Jean-Philippe Auger, Anish Pandey, Paddy Tighe, Myron Christodoulides

**Affiliations:** 1 Neisseria Research Group, Molecular Microbiology, Clinical and Experimental Sciences, Sir Henry Wellcome laboratories, University of Southampton Faculty of Medicine, Southampton General Hospital, Southampton, United Kingdom; 2 Department of Pathology and Microbiology, Faculty of Veterinary Medicine, University of Montreal, Saint-Hyacinthe, QC, Canada; 3 Queen's Medical Centre, Nottingham, NG7 2UH, United Kingdom; Centers for Disease Control & Prevention, UNITED STATES

## Abstract

*Streptococcus pneumoniae* (Spn) is a major causative organism of empyema, an inflammatory condition occurring in the pleural sac. In this study, we used human and Spn cDNA microarrays to characterize the transcriptional responses occurring during initial contact between Spn and a human pleural mesothelial cell line (PMC) *in vitro*. Using stringent filtering criteria, 42 and 23 Spn genes were up-and down-regulated respectively. In particular, genes encoding factors potentially involved in metabolic processes and Spn adherence to eukaryotic cells were up-regulated e.g. *glnQ*, *glnA*, *aliA*, *psaB*, *lytB* and *nox*. After Spn initial contact, 870 human genes were differentially regulated and the largest numbers of significant gene expression changes were found in canonical pathways for eukaryotic initiation factor 2 signaling (60 genes out of 171), oxidative phosphorylation (32/103), mitochondrial dysfunction (37/164), eIF4 and p70S6K signaling (28/142), mTOR signaling (27/182), NRF2-mediated oxidative stress response (20/177), epithelial adherens junction remodeling (11/66) and ubiquitination (22/254). The cellular response appeared to be directed towards host cell survival and defense. Spn did not activate NF-kB or phosphorylate p38 MAPK or induce cytokine production from PMC. Moreover, Spn infection of TNF-α pre-stimulated PMC inhibited production of IL-6 and IL-8 secretion by >50% (p<0.01). In summary, this descriptive study provides datasets and a platform for examining further the molecular mechanisms underlying the pathogenesis of empyema.

## Introduction

*Streptococcus pneumoniae* (Spn) is a major causative organism of non-invasive diseases such as otitis media and invasive diseases including pneumonia, meningitis, sepsis and empyema [1]. Empyema is a compartmentalised inflammatory response occurring in the pleural sac as a complication of bacterial pneumonia but the pathogenesis is poorly understood. The disease affects both children and adults and in children the most common causative agent is *S*.*pneumoniae* (60–70% of cases), although infection with *Staphylococcus aureus* and *Streptococcus pyogenes* and *Streptococcus milleri*, *Mycoplasma pneumoniae* and *Pseudomonas aeruginosa* has also been reported. In adults, the bacterial aetiology of empyema is more complex in both community- and hospital-acquired infections. The reported incidence and proportions of empyema has increased steadily from 1990 to 2012, even before introduction of the PCV-7 pneumococcal conjugate vaccine [2]. Predominant causes of empyema were identified as serotypes 1, 19A, 3, 14, and 7F and additionally serotype replacement and the emergence of non-vaccine serotypes following introduction of PCV-7 likely contribute to reported increases in empyema [3]. However, introduction of PCV-13, which includes many of the emergent serotypes associated with increased empyema, may impact on disease incidence.

Anatomically, the lungs are surrounded by two mesodermally-derived serous membranes called the pleurae, which are each composed of a monolayer of mesothelial cells, connected via tight junctions and these monolayers loosely overlay a basement membrane. Normally, pleural fluid within the cavity is sterile and contains ~1.7x 10^6^ cells per ml, comprised of resident macrophages/sentinel dendritic cells (DC) (75%), lymphocytes (23%), polymophonuclear cells (<3%) and free mesothelial cells (2%) [4]. Pathologically, Spn empyema is characterised by the formation of pus within the pleural sac and if untreated, purulation progresses to a fibrinopurulent stage with loculations and resolution with the deposition of a thick fibrin ‘rind’. In empyema patients, infected pleural fluid is characterised by the presence of inflammatory mediators including interleukin (IL-)1, IL-6, IL-8, monocyte chemoattractant protein (MCP)-1, tumour necrosis factor (TNF)-α and platelet activating factor [5,6]. These mediators likely contribute to increased blood vessel permeability, neutrophil, lymphocyte and eosinophil infiltration, fluid accumulation and activation of coagulation and fibrinolytic pathways. The morbidity of empyema is high and treatment involves antibiotic therapy and often surgical interventions such as chest drainage [2].

Understanding the pathogenesis of Spn empyema has benefited from the development of *in vivo* and *ex vivo* models of infection. Wilkosz and colleagues described a murine model that mimicked human pleural infection, whereby intranasal inoculation of the laboratory reference Spn strain D39 resulted in bacterial invasion of the pleural sac from the lung, followed by leucocytosis predominantly of neutrophils and monocytes, cytokine production (TNFα, MCP-1, IL-8/MIP-2 and VEGF) and the development of pleural fibrin depositions [6]. The murine model suggested that D39 pneumococci rapidly crossed the pleural layer from the lung parenchyma. The authors also showed that pneumococci adhered to an *in vitro* model of empyema based on human Met-5A pleural mesothelial cells (PMC) and subsequently invaded the mesothelial cells. However, neither the contribution of PMC to the inflammatory process nor the identification of specific Spn factors required for virulence, were reported. In the current study, we used a dual transcriptomics approach to map the patterns of host and pathogen gene expression during the early stages of infection of Met-5A cells with Spn *in vitro*. Our hypothesis was that the transcriptional landscape of PMC and pneumococcus during initial interactions of planktonic bacteria could inform the development of empyema and potentially present new targets for interventions at the level of both host cell and pathogen.

## Materials and Methods

### Culture and characterization of Met-5A cell line

The PMC line Met-5A (ATC-CRL-9444, LGC Standards, UK) was grown in Medium 199 containing Earles’ balanced salt solution, glutamine, HEPES and sodium carbonate (Lonza, UK), supplemented with 10% (v/v) decomplemented foetal bovine serum (dFBS), 3.3nM epidermal growth factor, 870nM insulin and 400nM hydrocortisone (Sigma-Aldrich, UK) [6]. Met-5A PMC cells were characterised by positive immuno-histochemical staining for cytokeratin, E-cadherin and calretinin [7] and for positive expression of Toll-Like Receptors (TLR)2 and TLR4 by flow cytometry (S1 Fig).

### Culture of *Streptococcus pneumoniae* and infection of Met-5A cells

Serotype 2 capsule polysaccharide expressing strain D39 (NCTC 7466) bacteria were grown on Columbia Blood Agar Plates (E&O Laboratories Ltd., UK),incubated overnight at 37°C and 5% (v/v) CO_2_ in the presence of optochin discs (Mast Group, UK) to confirm culture purity. For Met-5A infection experiments for microarray analyses, D39 was grown in liquid Brain Heart Infusion (BHI) medium (Oxoid, UK) to mid-log phase, the cultures were centrifuged (2500g, 10 min at room temperature (RT) and washed once in 10ml of warm phosphate buffered saline (PBS, pH7.4). The suspension was adjusted to a concentration equivalent to a multiplicity of infection (MOI) of ~200 bacteria per cell in Medium 199 containing 1% (v/v) dFBS, which was used to infect confluent Met-5A cell monolayers grown in 30×75 cm^2^ cell culture flasks for 2h at 37°C. Control, uninfected cells were maintained in medium alone.

### Recovery of prokaryotic and eukaryotic RNA

For bacterial RNA, the recovery method used and validated was based on the procedure described by Ryan *et al*. [8]. Infected cell monolayers were washed twice with PBS to remove any non-adherent bacteria and the monolayers were treated with 0.005% (v/v) trypsin-EDTA (Lonza) for 10 min at 37°C, in order to detach adherent bacteria, whilst leaving the Met-5A monolayers intact. Control bacteria were maintained in infection medium alone, washed with PBS twice and treated with trypsin solution similarly. Detached and control bacteria were collected by centrifugation (2000g, 5min), the pellets suspended in 700μl of RNA Protect Bacteria Reagent (Qiagen, UK) with incubation for 10 min at RT to stabilise the bacterial RNA. The suspension was centrifuged (2000g, 5 min) and the resulting pellet was suspended in proprietary lysis buffer RLT (Qiagen), transferred to Lysis Matrix B tubes (MP Biomedicals) and mechanically lysed using a Ribolyser (Biorad). This process disrupted >70% of pneumococci recovered from monolayers and bacterial RNA was extracted as described below. The infective dose required to obtain a sufficient yield of bacterial RNA for microarray analysis was determined by infecting pleural cells with various MOI (1–250). A recovery rate of >95% was achieved at a MOI ~200, whereas ≤13% of bacteria were recovered with MOI of ≤50. A MOI ~200 infecting 30×75cm^2^ cell monolayers was required to yield at least 2μg of total pure bacterial RNA needed for microarray analysis and an infection time of 2h was chosen to examine initial interactions and because high infective doses of pneumococci induced cell death at later time points. To confirm that trypsin detachment had no effect on pneumococcal gene transcription, a bacterial microarray control experiment was done to compare RNA from trypsin-treated pneumococci with RNA from no treatment bacteria. Comparison of gene expression was done by ‘dye swap’ analysis of microarray slides as described below. In agreement with Ryan *et al*. [8] the microarray data for gene expression of trypsin-treated pneumococci and untreated pneumococci were similar (personal communication, Jason Hinds, St. George’s Hospital, London), thus validating trypsin detachment as a suitable method for bacterial transcriptome analysis.

After detachment of pneumococci, Met-5A infected and control (uninfected and trypsin-treated) cell monolayers were disrupted by the addition of lysis buffer RLT (700μl/flask). Bacterial and host cell RNA were extracted from their respective lysates using the RNeasy Minikit (Qiagen, UK). Concentrations of RNA and purity of samples were quantified on a Nanodrop® ND1000 spectrophotometer (Nanodrop®, USA). Homogeneity of the respective RNA samples and the absence of human RNA contamination of bacterial samples was confirmed by sample analysis on a Bioanalyser 2100 (Agilent Technologies, USA) using an RNA Nano Kit 6000, following the manufacturer’s instructions. RNA Nanochips were analysed on the Bioanalyser using the manufacturer’s Prokaryote Total RNA Nano protocol.

### Labelling of *S*.*pneumoniae* RNA and microarray hybridization

Cy3 and Cy5 (GE Healthcare) fluorochrome-labelled cDNA samples were prepared for each RNA preparation and were purified using MinElute columns (Qiagen). The SPv2.0.0 microarrays used in this study were manufactured by the Bacterial Microarray Group at St. George’s Hospital, University of London as oligonucleotide (60mer) microarrays, designed to sequences from EnsemblBacteria version 5 (http://bacteria.ensembl.org). The arrays were based on the entire TIGR4 genome sequence [9] extended by additional genes from other pneumococcal strains [10] and included 2353 target genes in total. Microarray slides were incubated in a pre-hybridisation solution (20x saline sodium citrate (SSC), 20% (w/v) sodium dodecyl sulphate (SDS), 100mg/ml bovine serum albumin (BSA) for 65°C for 20 min.), rinsed with water and 100% propanol, dried and stored in the dark (for <1h) in a dust-free box.The pre-hybridised slide was placed into a hybridization cassette (Corning Lifesciences) and purified Cy3/Cy5-labelled cDNA was added to a hybridization solution (20x SSC, 2% (w/v) SDS), heated to 96°C for 2 min., briefly cooled and then pulse centrifuged. A LifterSlip (VWR International) was placed over the printed area of the array slide and the hybridization solution introduced by capillary action across the array. The hybridization cassette was sealed and submerged in a water bath at 65°C in the dark for 16-20h. The microarray slide was then removed from the hybridization cassette, washed in 20x SSC, 2% (w/v) SDS buffer to remove the LifterSlip, washed in 0.06x SSC buffer and centrifuged (500g for 5 min) to thoroughly dry. Slides were scanned on an Affymetrix GeneChip analyser (Affymetrix, USA). Differentially expressed pneumococcal genes were identified using BlueFuse for Microarrays 3.5© software (BlueGnome Ltd., UK), a local weighted scatterplot smoothing (LOWESS) normalization algorithm and filtering algorithms in GeneSpring GX v7.3.1 software (Agilent, USA).

### Analysis of gene expression profile of human Met-5A cells

#### i) Human Microarrays

Consised of 32,488 human genes, printed in-house at the Post-Genomic Technologies Facility at the University of Nottingham (http://genomics.nottingham.ac.uk) using a BioRobotics Microgrid II 600 robot (Isogen Life Science, Netherlands). Synthesized cDNA probes were spotted onto the surface of Nexterion A+ slides (Schott, UK) and all slides were scanned using an Agilent BA scanner to detect auto-fluorescence and precisely locate each spot. Each image was processed using GenePix® Pro v6.0 software (Molecular Devices LLC, USA) and a tolerance threshold of <1% maximum missing spots (<320 out of 32488) was applied to all arrays.

To ensure homogeneity between spots, cDNA distribution within each spot was quantified on two slides from both the beginning and end of each print run. The printed arrays, as well as a proprietary calibrator slide, were hybridized with Genetix Spotcheck™ solution (Molecular Devices) and scanned at λ_555_nm. The signal intensities from the spots on the printed slide were compared to those on the control slide and the relative quantity of cDNA was calculated by the manufacturer’s quality control software (Agilent Technologies). Spots with signal intensities below that of the lowest calibrator were excluded from analysis.

To negate any error arising from inter-batch variability, all slides originated from the same print run. An additional two slides were assessed using the Lucidea Microarray ScoreCard system (Amersham Biosciences, USA). A set of 10 artificial genes, selected from yeast intergenic regions that show no cross-hybridisation with human, mouse, rat or *E*. *coli* were also printed onto each array and hybridised to corresponding control Cy3/Cy5-labelled probes. Lucidea Microarray ScoreCard software was used to evaluate target attachment, probe labelling efficiency, hybridisation uniformity, signal detection limits and dynamic range of each slide and apply quality control criteria. Comparison of these parameters, both intra- and inter- experimentally, also provided a method of data normalisation.

#### ii) Synthesis and purification of human cDNA and in-vitro transcription to yield amino-allyl RNA (aRNA)

All reagents, consumables and instructions for preparing human microarray probes were provided in an Amino Allyl MessageAmp™ II aRNA proprietary kit (Ambion, USA). cDNA first and second strands were synthesized and purified cDNA was transcribed in the presence of amino-allyl labelled uracil triphosphate (aaUTP) to yield amino-allyl- labelled RNA (aRNA), which was purified using a manufacturer’s proprietary filter cartridge. Incorporation of aaUTP was confirmed by measurement on a Nanodrop spectrophotometer. Test aRNA samples were dye-coupled to Cy3, whilst control aRNA was labelled with Cy5 and dye-labelled aRNA samples were purified using aRNA filter cartridges and the frequency of dye incorporation was measured on the Nanodrop spectrophotometer. For each slide to be hybridized and prior to use, 500ng of both test sample and control sample aRNA was fragmented using proprietary fragmentation buffer (Ambion). Samples were placed on ice and 2μl of lithium heparin and 1μl of Cot-I human DNAse (Ambion) was added to inhibit non-specific probe hybridization, followed by pre-heated (50°C) Schott 1X hybridisation buffer, prior to slide hybridization.

#### iii) Microarray slide hybridisation and data analysis

Microarrays were washed sequentially with 0.2% (w/v) SDS buffer, PBS containing 0.1M sodium borohydride, 0.2% SDS (w/v), water and ethanol (100%). Microarray hybridizations were done on a HS48000 hybridization station (Tecan Ltd., Switzerland), using an automated program and 0.1–4 X SSC/0.1–0.2% (w/v) SDS buffer solutions as recommended by the manufacturer.Slides were scanned on an Agilent BA scanner and two digital images were obtained for each array, which were overlaid using the manufacturer’s software. Images were exported to and analyzed using GenePix® Pro v6.0 software (Molecular Devices LLC, USA) and the median of duplicate spots was calculated for each image and raw data were expressed as a log ratio of Cy5/Cy3 (sample/control probe). Intra- and inter-array global LOWESS normalization was done in J- Express Pro software (MolMine, Norway) and ratios were then used to rank genes and calculate fold changes. A False Discovery Rate (FDR) limit of ≤ 5% was applied to the gene lists and genes with an FDR above this cut-off were excluded from analysis [11].

### Examination of pneumococcal and human mRNA transcription by reverse transcription (RT)-qPCR

Bacterial samples were collected as described above and a two-step RT-qPCR method was used to measure pneumococcal mRNA transcription of selected genes using 18-20bp primers (S1 Table). Test and control RNA were extracted as described above and cDNA was prepared using the Superscript® VILO™ cDNA Synthesis Kit (Invitrogen, UK). The relative expression levels of pneumococcal genes were analyzed using qPCR done in triplicate on an Applied Biosystems 7900HT Fast Real-Time PCR System. Reaction mixtures contained EXPRESS SYBR® GreenER™ qPCR Supermix Universal (10μl), 10μM forward primer (200nM final, 0.4μl), 10μM reverse primer (200nM final, 0.4μl), Template DNA (2.5μl) and Nuclease-free water (6.7μl). The optimized qPCR conditions were incubation at 50°C for 2 mins, 40 thermocycles of 95°C for 15 sec and 55°C for 1 min.

For human mRNA transcription, total RNA was extracted from infected and non-infected cell culture (n = 3 independent experiments) using the RNeasy Mini Kit (Qiagen). RNA samples were treated with DNAse-50 (PrimerDesign) and total RNA concentration was quantified using a NanoDrop spectrophotometer. Using the RT-nanoscript system (PrimerDesign) and the reverse oligonucleotides listed in S1 Table, 1 μg of RNA from infected and non-infected conditions was used to synthetize the corresponding cDNA for 21 human genes. RT-qPCRs were done using 1 μl of each cDNA in a Rotorgene-Q 5plex HRM (Qiagen) using the Power SYBR green PCR Master Mix (Life Technologies) and specific oligonucleotides (S1 Table).

Analysis of relative mRNA transcription was facilitated by normalization to transcription of *gyrA* for pneumococci and *GAPDH* for Met-5A cells, using the 2–ΔΔCt method. To ensure a single specific amplification product, melt curve analysis was done between 50–95°C using SDS v2.4 software; melting temperatures were calculated using the BioMath—Tm Calculator for Oligos (Promega). The unpaired one-tailed T-Test was used to test the statistical significance of the difference between cycle threshold (Ct) values and relative differences in fold expression.

### *S*.*pneumoniae* infection of Met-5A cells for quantifying cytokine secretion, NF-kB and MAP kinase p38 pathway activation and cell death

Triplicate confluent Met-5A cells (~4×10^5^ cells/monolayer) in 24 well cell culture plates (Greiner bio-One) were infected with different MOI (2.5× 10^−5^ to 250) of D39 bacteria in DMEM containing 1% (v/v) dFBS and incubated at 37°C and 5% (v/v) CO_2_. Adherence and invasion by Spn were quantified using the gentamicin-saponin lysis and viable counting methods as described previously [12]. In antibiotic sensitivity assays, this gentamicin dose killed >99.99% of extracellular pneumococci within 90min.

Mid-log phase cultures of pneumococci were inactivated by heating to 56°C for 30 min. To prepare pneumococcal lysates, mid-log phase suspensions were centrifuged and bacterial pellets transferred to Lysis Matrix B tubes and mechanically lysed on ice using a Ribolyser. A sterile filtrate was also isolated from the pneumococcal lysates by passing the lysate through a 0.2μm filter to remove bacterial debris and large polysaccharides. Viable counting confirmed that the preparations did not contain live pneumococci. Functionality of the pneumolysin protein in lysates was confirmed by erythrocyte haemolytic assay, as described previously [13]. Confluent Met-5A cells were treated with the crude lysate of mechanically disrupted cells, the sterile filtrate or heat-inactivated bacteria, each at a concentration of 100μg/ml as determined with a bicinchoninic acid assay (Pierce) as well as with *Neisseria meningitidis* (Nm)-outer membrane (OM) preparations [12] or pure TNFα (ebioscience; 30–100 ng/monolayer).

All supernatants were retained at 24h and Ready-SET-Go® ELISA kits (eBiosciences, USA) were used for cytokine quantification. Absorbance was measured on an iMark Absorbance Reader at λ490nm with λ570nm correction (Biorad) and the manufacturer’s software was used to derive cytokine standard curves and to calculate cytokine concentrations. NF-kB p65 and phosphor-p38 proteins were quantified at time points up to 2h after infection or addition of treatments using InstantOne ELISA kits (eBioscience). A two-sample t-Test was used to compare between treatments, with p values <0.05 considered significant.

A lactate dehydrogenase (LDH) cell viability assay was used to quantify cell death as described previously [13]. Briefly, Met-5A cells were grown to confluence in 96-well tissue culture plates and infected in triplicate with various MOI (2.5× 10^−5^ to 250) of the different pneumococcal strains. LDH release was measured at intervals up to 9h using the CytoTox 96® Non-Radioactive Cytotoxicity assay kit (Promega, UK). Absorbance was read at λ492nm with an iMark Absorbance Reader (Bio-Rad) and cytotoxicity levels were calculated by dividing the average LDH release value of test by the average Maximum LDH release value (from lysed, uninfected cells) using the following formula: LDH release of test (%) = (LDH release of test/Maximum LDH release) x100.

## Results

### Gene expression in *Streptococcus pneumoniae* following interaction with human Met-5A pleural mesothelial cells (PMC)

**Fig 1:**
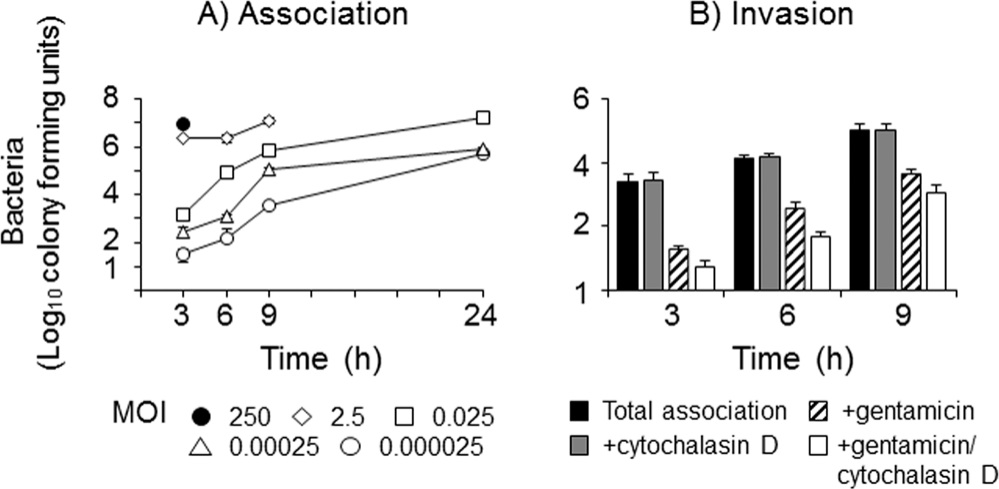
Interactions of *Streptococcus pneumoniae* with pleural mesothelial cells *in vitro*. A) Association: monolayers of Met-5A cells were infected with various MOI of *Streptococcus pneumoniae* strain D39. Total associated (defining adherent and internalised bacteria combined) bacteria were counted following saponin lysis of infected monolayers over time. The data are representative of >3 independent experiments; the symbols represent mean adherence and the error bars the standard deviations of wells infected in triplicate. B) Invasion: monolayers of Met5A cells were infected for 9h with MOI 0.025 of D39 and intracellular bacteria were recovered after gentamicin treatment and saponin lysis. Monolayers were also pre-treated with cytochalasin D prior to bacterial infection. The bars represent the mean bacterial cfu of three independent experiments and the error bars the standard error of the means.

Initially, the dynamics of D39 association with Met-5A cells and any subsequent invasion were quantified over time (Fig 1). High levels of saturating association of D39 were observed with initial infective MOI>2 by 3h, whereas with lower initial MOI ≤0.025, bacteria adhered in a dose and time-dependent manner. An initial MOI of 250 of D39 induced significant cytotoxicity by 6h, whereas the MOI of 2.5 only destroyed the monolayers by 24h. Bacteria could be recovered at 24h from intact monolayers infected with the lowest initial MOI tested (Fig 1A). Cell death following infection with D39 was measured by LDH release. Background levels (spontaneous) of LDH release by uninfected Met-5A cells were between 3–11%. Cell death induced by D39 that was visualized with high MOI (2–250) was not associated with significant LDH release; only 22% LDH release was detected following infection with the highest MOI tested (250) and the levels quantified after infection with MOI of <25 were not significantly different to background levels (p>0.05).

The ability of D39 to invade Met-5A cells was quantified using a standard gentamicin-saponin lysis assay. From preliminary titration experiments, an initial MOI of 0.025 of D39 was chosen to infect Met-5A cells to ensure sufficient bacterial association to allow quantification of invasion up to 9h without pleural cell death. There was no significant recovery of internalized D39 at 3h (<1% of internalized bacteria after gentamicin treatment/total associated bacteria). By 6-9h, following addition of gentamicin, ~2% of D39 bacteria were recovered as a percentage of total associated bacteria (p<0.05) (Fig 1B). When compared to treatment with gentamicin alone, pre-incubation of the monolayers with CD significantly reduced (p<0.05) bacterial counts of internalized D39, although it should be stressed that the numbers of recovered bacteria per monolayer were extremely low, e.g. equivalent to finding one D39 pneumococcus per 80 cells.

**Fig 2:**
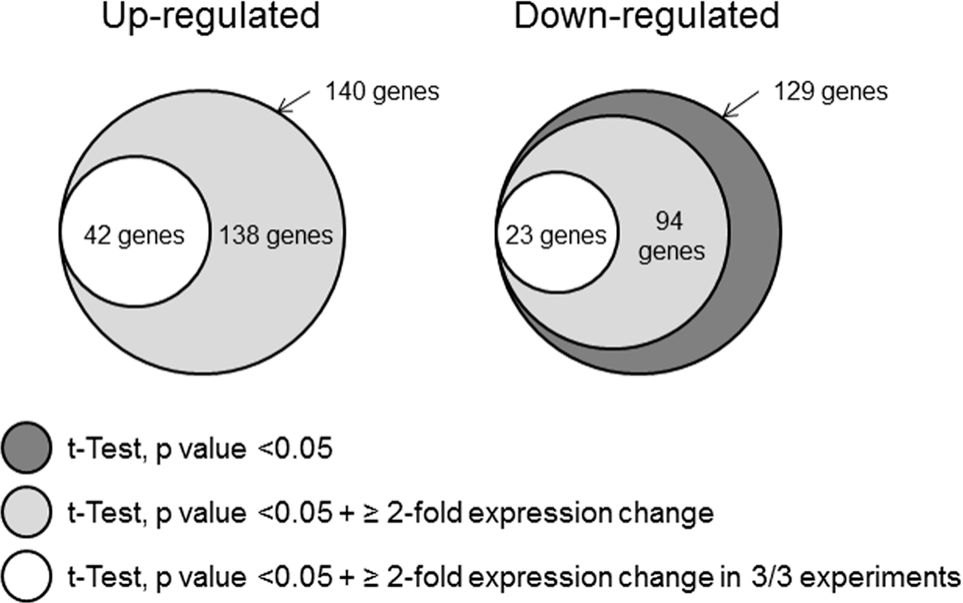
Differentially expressed pneumococcal genes identified by the application of data filtering algorithms. Gene lists generated by GeneSpring GX software were initially filtered using the students’ t-Test analysis of variance to yield only those genes with a p-value <0.05. Of these genes, only those displaying a mean ≥ 2-fold expression change were selected. Finally, only those genes meeting the filtering criteria in all biological replicates (n = 3) for a particular experimental condition were selected.

Gene expression in *S*.*pneumoniae* during initial contact with Met-5A cells was examined by microarray in three independent experiments following 2h of infection with initial MOI of 200, which was identified as the infection condition required to yield pure bacterial RNA needed for microarray analysis. Viable counting of adherent pneumococci estimated approximately 20 bacteria associated per cell. Comprehensive gene expression datasets for the individual experiments are shown in S2A Table, S2B Table. Stringent filtering criteria were applied to the microarray data (Fig 2) and 65 pneumococcal genes were identified in total as satisfying all of the criteria of the filtering algorithms, with 42 genes (64.6%) up-regulated (Table 1) and 23 genes (35.4%) down-regulated (Table 2).

**Table 1:** Up-regulated pneumococcal genes following adherence to human Met-5A PMC cells.

Gene	Gene Symbol	t-Test p- value	Mean Fold- Expression Change	Gene Product
*sptigr4-0136*	*sp_0136*	0.000156	3.6	glycosyl transferase, family 2
*sptigr4-0137*	*abc-np*	0.000447	4.7	ABC transporter, ATP-binding protein
*sptigr4-0138*	*sp_0138*	0.00055	3.6	hypothetical protein
*sptigr4-0139*	*sp_0139*	0.00604	4.0	hypothetical protein
*sptigr4-0140*	*ugd*	0.00117	5.4	UDP-glucose dehydrogenase
*sptigr4-0144*	*sp_0144*	0.000407	4.6	hypothetical protein
*sptigr4-0145*	*sp_0145*	0.000135	6.2	Hypothetical protein
*sptigr4-0231*	*adk*	0.00113	4.4	Adenylate kinase
*sptigr4-0366*	*aliA*	0.00586	5.7	oligopeptide ABC transporter, oligopeptide-binding protein AliA
*sptigr4-0445*	*ilvB*	0.00382	4.0	acetolactate synthase catalytic subunit
*sptigr4-0502*	*glnA*	0.00268	3.8	Glutamine synthetase, type I
*sptigr4-0636*	*abc-nbd*	0.000134	2.6	ABC transporter, ATP-binding protein
*sptigr4-0857*	*sp_0857*	0.000433	3.0	ABC transporter substrate-binding protein
*sptigr4-0858*	*sp_0858*	0.000227	2.4	Hypothetical protein
*sptigr4-0859*	*sp_0859*	0.00143	2.8	Membrane protein
*sptigr4-0862*	*rpsA*	0.00393	6.4	30S ribosomal protein S1
*sptigr4-0957*	*sp_0957*	0.000838	2.9	ABC transporter, ATP-binding protein
*sptigr4-0961*	*rplT*	0.00207	2.7	50S ribosomal protein L20
*sptigr4-0964*	*pyrD*	0.000401	2.8	Dihydroorotate dehydrogenase 1B
*sptigr4-0965*	*lytB*	0.00339	3.1	endo-beta-N- acetylglucosaminidase
*sptigr4-1241*	*glnP*	0.000721	8.8	Amino acid ABC transporter, amino acid-binding protein/permease protein
*sptigr4-1242*	*glnQ*	0.000302	10.0	Amino acid ABC transporter, ATP-binding protein
*sptigr4-1306*	*gdhA*	0.0000353	8.7	Glutamate dehydrogenase
*sptigr4-1468*	*pdx1*	0.00138	6.4	pyridoxine biosynthesis protein
*sptigr4-1469*	*nox*	0.000609	3.2	NADH oxidase
*sptigr4-1511*	*atpH*	0.000547	3.3	F0F1 ATP synthase subunit delta
*sptigr4-1512*	*atpF*	0.000216	2.6	F0F1 ATP synthase subunit B
*sptigr4-1513*	*atpB*	0.000988	3.3	F0F1 ATP synthase subunit A
*sptigr4-1537*	*sp_1537*	0.000447	2.7	hypothetical protein
*sptigr4-1538*	*sp_1538*	0.00409	4.2	Cof family protein/peptidyl-prolyl cis-trans isomerase, cyclophilin type
*sptigr4-1597*	*sp_1597*	0.000216	3.0	Hypothetical protein
*sptigr4-1598*	*pdxK*	0.000547	4.8	Phosphomethylpyrimidine kinase
*sptigr4-1599*	*truA*	0.000447	5.8	tRNA pseudouridine synthase A
*sptigr4-1648*	*psaB*	0.000216	4.0	Manganese ABC transporter, ATP- binding protein
*sptigr4-1715*	*abc-nbd*	0.000407	4.0	ABC transporter, ATP-binding protein
*sptigr4-1855*	*adhB*	0.00268	4.4	alcohol dehydrogenase, zinc- containing
*sptigr4-1856*	*sp_1856*	0.000227	3.6	Transcriptional regulator, MerR family
*sptigr4-1857*	*czcD*	0.0000353	4.9	Cation efflux system protein
*sptigr4-1913*	*sp_1913*	0.000245	4.8	Hypothetical protein, cspC-related protein
*sptigr4-1914*	*sp_1914*	0.0000353	5.5	Hypothetical protein
*sptigr4-1992*	*sp_1992*	0.000447	2.6	cell wall surface anchor family protein
*spr6-1178*	*spr1178*	0.00116	3.9	Hypothetical protein

Differential gene expression was analyzed by microarray in pneumococci adherent to Met-5A cells at 2h post challenge. Genes demonstrating ≥ mean 2-fold changes in expression in all (n = 3) replicates with significance (t-Test p-value) are ordered by *sp* number.

**Table 2:** Down-regulated pneumococcal genes following adherence to human Met- cells.

Gene	Gene Symbol	t-Test p-value	Mean Fold- Expression Change	Gene Product
*sptigr4-0033*	*sp_0033*	0.00993	3.4	hypothetical protein
*sptigr4-0045*	*purL*	0.00451	2.2	phosphoribosylformylglycinamidine synthase, putative
*sptigr4-0046*	*purF*	0.0154	2.2	Amidophosphoribosyltransferase
*sptigr4-0092*	*abc- sbp*	0.0309	3.0	ABC transporter, substrate-binding protein
*sptigr4-0207*	*sp_0207*	0.0233	2.9	hypothetical protein
*sptigr4-0285*	*adhP*	0.00895	9.2	alcohol dehydrogenase
*sptigr4-0317*	*kdgA*	0.00531	4.6	keto-hydroxyglutarate- aldolase/keto-deoxy- phosphogluconate aldolase
*sptigr4-0320*	*gno*	0.0068	8.4	Gluconate 5-dehydrogenase
*sptigr4-0324*	*pts-eii*	0.00501	5.2	PTS system, IIC component
*sptigr4-0999*	*ccdA*	0.00501	2.6	cytochrome c-type biogenesis protein CcdA
*sptigr4-1069*	*abc- sbp*	0.00727	2.6	hypothetical protein
*sptigr4-1070*	*abc- msp*	0.0158	2.2	hypothetical protein
*sptigr4-1090*	*sp_1090*	0.00642	3.7	redox-sensing transcriptional repressor Rex
*sptigr4-1193*	*lacA*	0.0119	5.0	Galactose-6-phosphate isomerise subunit LacA
*sptigr4-1775*	*sp_1775*	0.0309	3.4	hypothetical protein
*sptigr4-1776*	*trxA*	0.0108	3.9	Thioredoxin
*sptigr4-1815*	*trpD*	0.0466	7.8	Anthranilate phosphoribosyltransferase
*sptigr4-1834*	*ptcC*	0.00022	2.1	PTS system, IIC component
*sptigr4-1859*	*sp_1859*	0.00267	5.7	transporter, putative
*sptigr4-1988*	*sp_1988*	0.00304	2.3	immunity protein, putative
*sptigr4-2026*	*adhE*	0.00407	28.4	alcohol dehydrogenase,
*sptigr4-2184*	*glpF*	0.0135	2.5	glycerol uptake facilitator protein
*sptigr4-2187*	*sp_2187*	0.0225	3.2	hypothetical protein

Differential gene expression was analyzed by microarray in pneumococci adherent to Met-5A cells at 2h post challenge. Genes demonstrating ≥ mean 2-fold changes in expression in all (n = 3) replicates with significance (t-Test p-value) are ordered by *sp* number.

### Expression of pneumococcal genes up-regulated in all independent experiments

The highest up-regulated genes in pneumococci following adherence to PMC were associated with glutamine metabolism, *i*.*e*. the *gdhA* gene encoding glutamate dehydrogenase, the *glnP* and *glnQ* genes that encode the major pneumococcal glutamine/glutamate transporter and the *glnA* gene encoding glutamine synthase (9–10 fold; Table 1). These genes are under the control of the transcriptional regulator *glnR* in the pneumococcus [14]. Examples of other genes involved in metabolic processes include the *aliA* gene, encoding the oligopeptide ABC transporter, oligopeptide binding protein, which exhibited ~6-fold increase in expression. Expression of surface antigen *psaB* gene, which is involved in manganese (Mn^2+^) transport, and the *czcD* gene that encodes a zinc efflux protein, exhibited 4–5 fold increases in expression in pneumococci adherent to PMC. A pneumococcal ABC transporter gene (*abc-nbd*, *spd-0636*), was up-regulated also in all three biological replicates (mean fold increase ~3). Up-regulation by 5-fold of expression of the *ugd* gene, encoding a UDP-glucose dehydrogenase essential for capsule biosynthesis, was also observed. Expression of the *lytB* gene that encodes for a murein hydrolase essential for cell separation [15] and *pyrD* gene, which is located downstream of *lytB* and possibly forms part of the same transcriptional unit, was up-regulated 3-fold.

Up-regulated gene expression was observed for other enzymes involved in key metabolic processes, including *ilvB* [16], *pdxK*,*pdX1*, *sp_0136*, *sp_1855* (*adhB*) encoding an alcohol dehydrogenase, as well as genes encoding ribosomal proteins *rplT*,*rpsA*, tRNA processing (*truA*), ABC-transporter-peptidyl prolyl trans-isomerisation (*sp_1538*) and a *csp-C* related gene product (*sp_1913*) that is required for competence and has been observed up-regulated in mouse blood infected with D39 [17]. Upregulated gene expression was observed for the *nox* gene encoding an NADH oxidase (3-fold), for the *adK* gene encoding adenylate kinase (4-fold) and for the F0F1 ATPase related genes *atpB*, *atpF* and *atpH* (3-fold). In addition, expression of several hypothetical gene products of unknown function, *e*.*g*. *sp_0145*, *sp_1856*, *sp_1715*,and other ABC-transporter proteins were significantly up-regulated in D39 following initial interactions (Table 1).

### Expression of pneumococcal genes down-regulated in all independent experiments

The genes *adhE* and *adhP*, encoding for alcohol dehydrogenases, showed the highest mean fold down-regulation (28- and 9-fold respectively) in expression (Table 2). Genes involved in the phosphoenolpyruvate-dependent (PEP) phosphotransferase system (PTS, i.e. *pts-eii*, *ptcC*) were also down-regulated in D39, which may possibly be a consequence both of glucose depletion and the lack of hyaluronic acid as a carbon source in infection medium [18]. Other down-regulated genes included a putative immunity protein (*sp_1988*) and several hypothetical proteins of unknown function (Table 2).

Changes in pneumococcal gene expression on interaction with Met-5A cells as identified by microarray analyses were validated by selecting pneumococcal genes for RT-qPCR using the random stratification method [19]. Gene expression was determined for 24 genes (Table 3), of which 15 genes showed up-regulated expression by microarray and the remaining 9 showed down-regulation. Significant increases in gene expression were observed by RT-qPCR for the microarray up-regulated genes *psaB*, *gdhA*, *glnQ*, *sp_0858*, *pdx1 and sp_1992* (p<0.05), marginally significant for *sp_1914* (p = 0.06) and insignificant for the remaining eight genes (p>0.05). For the genes that showed down-regulated gene expression by microarray, RT-qPCR identified significant down-regulated gene expression for *sp_1775*, *abc-sbp*, *adhP*, *purF*, *purL* and *adhE* (p<0.05) and insignificant for the remaining three genes (p>0.05).

**Table 3:** mRNA transcription by RT-qPCR for selected pneumococcal genes.

Gene	Genesymbol	Mean foldChange inmRNA—geneexpression	T-Testp value	Mean fold changein gene expressionby microarray	Gene product
				*Up-regulated*	
*sptigr4-0136*	*sp_0136*	2.0	0.14	3.6	Glycosyl transferase, family 2,putative
*sptigr4-0138*	*sp_0138*	2.6	0.17	3.6	Hypothetical protein
*sptigr4-0502*	*glnA*	4.0	0.5	3.8	Glutamine synthetase, type I
*sptigr4-0857*	*sp_0857*	2.7	0.16	3.0	Hypothetical protein
*sptigr4-0858*	*sp_0858*	41.3	0.03*	2.4	Membrane protein, putative
*sptigr4-0965*	*lytB*	1.7	0.18	3.1	Endo-beta-N-acetylglucosaminidase
*sptigr4-1242*	*glnQ*	3.9	0.03*	10.0	Amino acid ABC transporter, ATP-binding protein
*sptigr4-1306*	*gdhA*	4.5	0.03*	8.7	NADP-specific glutamate dehydrogenase
*sptigr4-1468*	*pdx1*	3.9	0.05*	6.4	Pyridoxine biosynthesis protein
*sptigr4-1469*	*noxR*	1.3	0.22	3.2	NADH oxidase
*sptigr4-1598*	*pdxK*	1.3	0.14	4.8	Phosphomethylpyrimidine kinase
*sptigr4-1648*	*psaB*	1.9	0.02*	4.0	Manganese ABC transporter, ATP-binding protein
*sptigr4-1914*	*sp_1914*	24.5	0.06	5.5	Hypothetical protein
*sptigr4-1992*	*sp_1992*	12.6	0.05*	2.6	Cell wall surface anchor family protein
*spr6-1178*	*spr_1178*	62.6	0.21	3.9	Hypothetical protein
				*Down-regulated*	
*sptigr4-0045*	*purL*	8.3	0.03*	2.2	Putative Phosphoribosylformylglycinamidine synthase
*sptigr4-0046*	*purF*	6.3	0.03*	2.2	Amidophosphoribosyltransferase
*sptigr4-0285*	*adhP*	5.6	0.02*	9.2	Alcohol dehydrogenase
*sptigr4-1069*	*abc-sbp*	7.2	0.04*	2.6	Hypothetical protein
*sptigr4-1775*	*sp_1775*	33.6	0.02*	3.4	Hypothetical protein
*sptigr4-1776*	*trxA*	778	0.22	3.9	Thioredoxin
*sptigr4-1815*	*trpD*	83.1	0.12	7.8	Anthranilate phosphoribosyltransferase
*sptigr4-2026*	*adhE*	7.2	0.04*	28.4	Aldehyde-alcohol dehydrogenase
*sptigr4-2187*	*sp_2187*	2.15	0.18	3.2	Hypothetical protein

Using the random stratification method, differentially expressed genes were stratified and genes then randomly selected from each stratum for validation. Both up- and down-regulated genes lists were divided into strata containing three genes and random number generating software (Apple Inc.) was used to select the gene for validation. The data are from five independent infection experiments and three control samples, i.e. pneumococci grown without adherence to cells. RT-qPCR was done in triplicate and fold expression changes for each gene by microarray analysis are shown alongside. To demonstrate that possible contamination with host RNA did not impact on pneumococcal RT-qPCR mRNA expression data, as a control, RT-qPCR was also done using Met-5A PMC uninfected cDNA; for all genes examined, the level of non-specific amplification of Met-5A cell cDNA was <0.005% of the specific amplification of control pneumococcal cDNA.

* denotes statistical significance (Ct values) <0.05.

### Gene expression in human Met-5A pleural mesothelial cells (PMC) following interactions of *Streptococcus pneumoniae*

**Fig 3:**
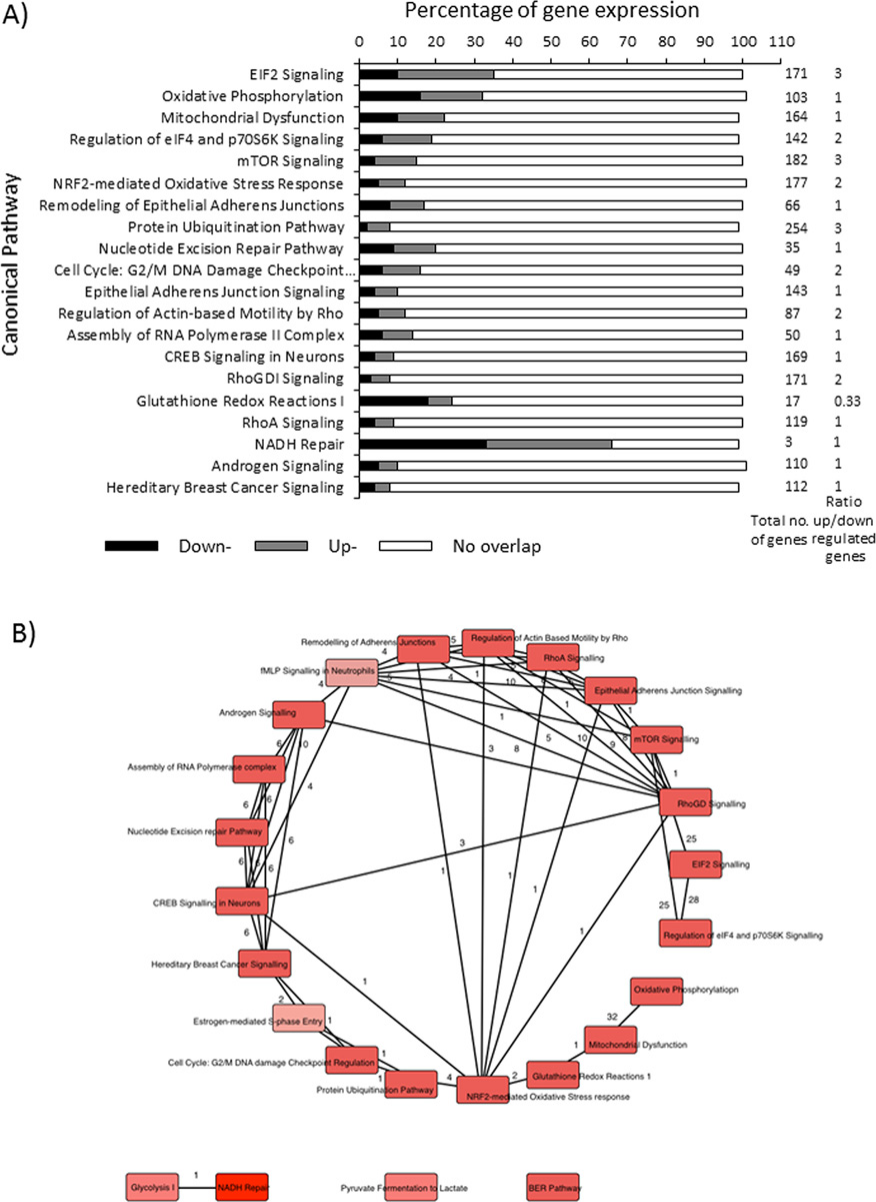
Analysis of data obtained from human microarray experiments using Ingenuity Pathways Analysis (IPA). A) IPA software v7.6 (Ingenuity® Systems, USA) was used to identify the top canonical pathways in PMC that were most affected by D39 infection. This was determined by calculating the ratio of the total number of differentially expressed genes in each pathway as a proportion of the total number of genes involved in that pathway. A minimum threshold of 5% gene perturbations was then imposed on pathways and those meeting these inclusion criteria were then ranked according to the proportion of gene perturbations. The 20 most highly ranked pathways by P-value and ratio are shown. B) Network map showing the distributions of specific genes that overlap between the top canonical pathways. The reader is referred to S3B Table for a complete listing of canonical pathways.

Gene expression in Met-5A PMC following pneumococcal interaction was examined using human microarrays in the same infection experiments and a comprehensive dataset of 870 genes is shown in S3A Table (for the average of n = 5 experiments). The data were analyzed using Ingenuity Pathways Analysis (IPA) software in order to identify the canonical pathways perturbed by D39 interaction and the ratios of up-and down-regulated genes (S3B Table). The top 20 canonical pathways showing the largest numbers of significant gene expression changes are shown in Fig 3A and included eukaryotic initiation factor (EIF)2 signaling (60 genes out of 171), oxidative phosphorylation (32/103), mitochondrial dysfunction (37/164), regulation of eIF4 and p70S6K signaling (28/142), mTOR signaling (27/182), NRF2-mediated oxidative stress response (20/177), remodeling of epithelial adherens junctions (11/66) and the ubiquitin pathway (22/254). Calculation of the ratio of the numbers of up- over down-regulated genes within the 20 canonical pathways demonstrated that there was a ~3-fold up-balance in the ratio of genes in the mTOR Signalling, Protein Ubiquitination Pathway and EIF2 Signalling pathways and a ~3-fold down-balance in the ratio of genes in the Glutathione Redox Reactions 1 pathway (Fig 3A). A further five pathways showed a ~2-fold up-balance in the ratio of gene expression and for the remainder the numbers of up- and down-regulated genes were similar (ratio of 1) (Fig 3A). IPA analysis also enabled the relationships between the canonical pathways to be plotted and Fig 3B shows a network map of the distributions of genes that overlap between the top canonical pathways.

In addition to identifying the canonical pathways, the ten individual most up-and down-regulated genes identified by microarray analysis were selected for validation by RT-qPCR (Table 4). D39 interaction induced up-regulated expression in PMC for the gene encoding the diazepam binding inhibitor protein (5-fold increase for *DBI*), and optineurin (4-fold increase for *OPTN*). Increased expression (~4-fold) was also quantified for *CNOT6*, *ATF4* and *NCL g*enes and the connective tissue growth factor *CTFG* gene (3-fold) as well as for genes encoding ribosomal proteins, transcriptional factors and the chaperone Hsp40 protein (Table 4). D39 infection down-regulated expression of the nuclear oncogene *SET* (~9-fold decrease), and also down-regulated expression ~5-fold of the genes *HNRNPC* (encoding the apoptosis-related heterogenous nuclear ribonucleoprotein C), *OAZ1* (encoding the apoptosis inducing ornithine decarboxylase antizyme 1), *SNRPB* (encoding the small nuclear ribonucleoprotein polypeptides) and *BST2* (encoding the bone marrow stromal cell antigen, tetherin). The majority of these Met-5A genes showing up-regulated expression following initial pneumococcal interactions also showed significant 2–3 fold-increases (p<0.05) in RT-qPCR validated measurement of gene expression, except for genes *OPTN*, *DNAJB6* and *CTGF* (Table 4). Using the microarrays, significant down-regulated expression of the genes *EEF1A1* (6-fold; encoding the translation regulatorEEF1A) and *GPX4* (~5-fold; encoding glutathione peroxidase) was observed. In addition, D39 infection down-regulated expression of the S phase-specific cell cycle thymidine kinase 1 (*TK1*) gene and the *ACTB* gene (~4-fold) encoding β-actin and the ADP-ribosylating factor *ARF4* gene (~4-fold) (Table 4). RT-qPCR validation showed that for all of the down-regulated targets, gene expression in infected cells was ≤2 fold lower compared with uninfected cells (p<0.05) (Table 4), except for *GPX4* (p = 0.07).

**Table 4:** RT-pPCR validation of microarray gene expression changes in human Met-5A pleural mesothelial cells following infection with D39 *Streptococcus pneumoniae*.

**Up-regulated gene expression**						
**Gene Symbol**	**Entrez Gene Name**	**Microarraygene expression fold-change**	**RT-qPCR gene expression fold-change**	**P-value (RT-qPCR validation)**	**Subcellular localisation**	**Function / Role in cellular processes**
*DBI*	Diazepam binding inhibitor(GABA receptor modulator, Acyl-CoA binding protein)	5.2	2.3	1.48e-0.7*	C	Acyl–CoA binding, benzodiazepine receptor binding.Role in proliferation and mitogenesis
*OPTN*	Optineurin	3.8	3.3	0.2346	C	Function in replication and cell death
*RPL6*	Ribosomal protein L6	3.8	1.8	1.41e-06*	R	DNA binding, RNA binding, structural component of ribosome. Role in translation.
*EIF4G2*	Eukaryotic transcription-initiationfactor 4 γ 2.	3.7	2.5	0.01*	C	Nucleic acid binding translation factor, protein binding, translation initiation factor, translation regulator. Function intranslation, cell cycle progression, cell death, growth,transformation, apoptosis, shunting, morphology,differentiation.
*SRSF4*	Serine/arginine-rich splicing factor 4	3.7	3.0	4.06e-10*	N	Nucleotide binding, RNA binding. RNA splicing.
*DNAJB6*	DnaJ (Hsp40) homologue,subfamily B, member 6	3.6	1.1	0.22	C/N	ATPase stimulator, chaperone binding, DNA binding,heat shock protein binding, transcription regulator. Active in cell death, organization, growth, formation.
*NCL*	Nucleolin	3.6	2.4	1.46e-12*	N	Nuclear localisation sequence, RNA binding, telomeric DNAbinding.Role in proliferation, apoptosis, synthesis, maturation, migration,macropinocytosis, transduction.
*CNOT6*	CCR4-NOT transcription complex,subunit 6	3.5	1.8	4.60e-7*	N	Transcription
*ATF4*	Activating transcription factor 4	3.5	1.5	0.001*	N	DNA binding transcription factor and transcription regulator.Expressed in apoptosis, cell death, cell cycle progression and isrecruited in the endoplasmic reticulum stress response.
*CTGF*	Connective tissue growth factor	3.4	2.1	0.06	ES	Fibronectin, heparin and integrin binding protein. Expressed in cell-cell adhesion processes, cell proliferation and growth.
**Down-regulated gene expression**						
**Gene Symbol**	**Entrez Gene Name**	**Gene expression fold-change**	**mRNA expression fold-change**	**P-value (RT-qPCR validation)**	**Subcellular localisation**	**Function / Role in cellular processes**
*SET*	SET nuclear oncogene	8.5	1.4	2.25e-06*	N	Histone binding, nuclear localisation sequence, phosphatise.Role in apoptosis, cell disassembly, cell death.
*BST2*	Bone marrow stromal cell antigen 2	7.9	1.3	3.18e-0.9*	PM	Signal transducing transmembrane protein, protein ubiquitination.
*EEF1A1*	Eukaryotic translation elongationfactor 1 α 1	6.1	1.1	2.57e-07*	C	GTP binding, protein-synthesising GTPase, translation regulator.Active in proliferation and cell death.
*HNRNPC*	Heterogeneous nuclearribonucleoprotein C	5.3	1.1	6.85e-11*	N	mRNA binding, nuclear localisation sequence. Functionalduring transcription, cell differentiation and apoptosis
*OAZ1*	Ornithine decarboxylase antizyme 1	5.2	1.2	0.05*	C/Cy	Ornithine decarboxylase inhibitor. Role in cell death and cytostasis.
*GPX4*	Glutathione peroxidase 4	5.1	0.9	0.07	C	Regulator of apoptosis, cellular organization, cell integrity, motility,damage, cell viability.
*TK1*	Thymidine kinase 1	4.6	1.0	0.02*	C/Cy	Zinc ion binding. Phosphorylated during apoptosis and necrosis.
*ACTB*	Actin, β	4.3	1.5	2.77e-06*	C	Structural constituent of cytoskeleton. Important for cellmorphology, motility, formation, endocytosis, apoptosis, chemotaxis, growth,constriction, cell spreading, pathogen entry.
*SNRPB*	Small nuclear ribonucleoproteinpolypeptides B and B1	4.2	2.4	0.0002*	C	RNA splicing
*ARF4*	ADP-ribosylation factor 4	4.1	1.2	3.29e-05*	C	GTPase, transport.

Met-5A cells were infected with D39 (MOI 200 bacteria/cell) for 2h. RNA was extracted from pleural cells and analyzed on human microarrays. Mean fold expression changes were calculated for each gene from n = 5 experiments and a ≥ 2-fold increase/decrease was considered significant. RT-qPCR validation was done from n = 3 independent infection experiments in triplicate. The 10 most differentially up- and down-regulated genes are shown, as derived from IPA analysis, and the remainder can be seen in the gene datasets in S3A and S3B Table. C, cytoplasm, Cy, cytosol; ES, extracelular space; PM, plasma membrane; N, nucleus; R, ribosomes.

* denotes statistical significance (Ct values) <0.05.

### Pneumococcal interactions with Met-5A PMC do not induce inflammatory signals

**Fig 4:**
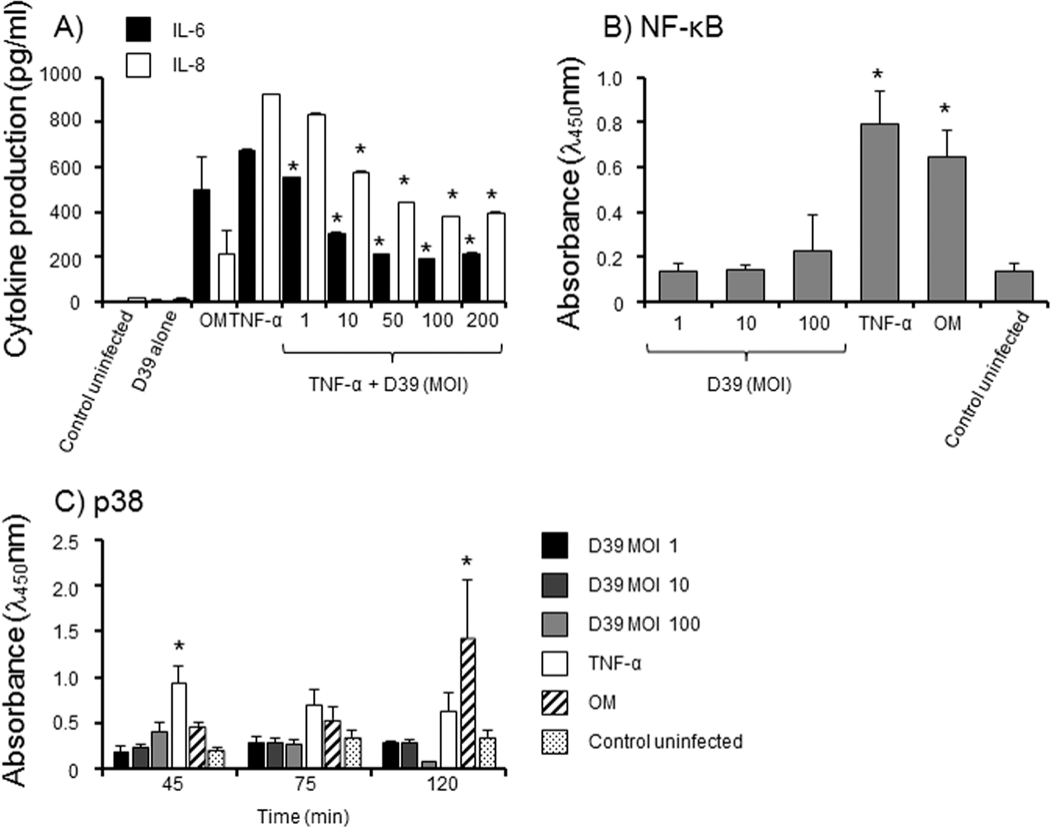
Inflammatory cytokine responses of Met-5A PMC. A) Met-5A cell monolayers (n = 3 wells) were infected in triplicate wells with live D39 (MOI 1–200), stimulated with pure TNF-α (100ng/ml) or Nm-OM (100ng/ml) and also pre-stimulated with TNF-α (100ng/ml) for 4h and then infected with various MOI (1–200) of live D39. Control cells were left with medium alone. Cells were also treated with live D39 without any other stimulation (no cytokine production at any of the concentrations tested between MOI 1–200). After 24h, supernatants were removed and production of IL-6 and IL-8 quantified by ELISA. The bars represent mean cytokine levels and the errors bars the standard deviation from triplicate wells from a representative experiment (n = 2). * denotes statistically significant reduction in IL-6 or IL-8 compared to cells treated with TNFα only (p<0.05). B) Met-5A cells were challenged in triplicate wells with live D39 bacteria (1–100 MOI), TNFα (30 ng/monolayer) or Nm-OM (30ng/monolayer). At intervals up to 2h, cells were processed and NFkB p65 protein measured by ELISA. The columns and symbols denote the mean absorbance readings from n = 3 experiments and the error bars the standard errors of the mean. * denotes statistically significant increase compared to control cells (p<0.05). C) Met-5A cells were challenged in triplicate wells with live D39 bacteria (1–100 MOI), TNFα (30 ng/monolayer) or Nm-OM (30ng/monolayer). At intervals up to 2h, cells were processed and p38 MAP Kinase protein measured by ELISA. The columns and symbols denote the mean absorbance readings from n = 3 experiments and the error bars the standard errors of the mean. * denotes statistically significant increase compared to control cells (p<0.05).

In the current study, microarray gene expression for inflammatory cytokines was not observed, which was confirmed by the absence of cytokine proteins in culture supernatants from PMC infected for 24h with live D39 (MOI ranging from 2.5× 10^−5^ to 250). There was no significant production of IL-6, TNF-α, IL-1β, IL-8, IL-10 or TGF-β, compared to control, uninfected cells (P>0.05). Furthermore, treatment of Met-5A cells with a whole crude lysate from mechanically lysed D39 and a sterile filtrate, which both contained active pneumolysin as demonstrated by erythrocyte haemolysis, did not induce significant cytokine production, compared to untreated cells (p>0.05). PMC were capable of producing cytokines, but only a high concentration of heat-inactivated bacteria (100μg/monolayer equivalent to a MOI of ~450) was capable of inducing significant IL-6 (~500 pg/ml) and IL-8 (~565 pg/ml) secretion (p<0.05) by Met-5A cells. PMC responded to Nm-OM and pure TNF-α alone with similarly detectable levels of IL-6 and IL-8 secretion (p<0.05) (Fig 4A).

We tested the hypothesis that D39 could inhibit cytokine production by pre-stimulating cells with TNF-α for 4h, then infecting with various doses of strain D39 and examining IL-6 and IL-8 production at 24h. D39 infection of TNF-α pre-stimulated PMC abrogated the production of both cytokines in a MOI-dependent manner (Fig 4A). TNF-α stimulated IL-6 secretion was significantly inhibited (p<0.01) by 55–72% with MOI from 10–200 and IL-8 by 50–60% (p<0.002) with MOI from 50–200. This inhibition was not due to degradation of TNF-α cytokine by D39 bacteria. Infection with D39 (1–100 MOI) did not induce significant (p>0.05) NF-κB activation, compared to activation with TNFα or Nm-OM (p<0.05) (Fig 4B). In addition, D39 infection did not induce significant phosphorylation of p38 MAP kinase (MAPK) (p>0.05), whereas significant (P<0.05) phosphorylation of p38 was induced by TNFα as early as 45min and a maximal response was observed by 2h with Nm-OM treatment (Fig 4C).

## Discussion

The current study is a descriptive analysis of the gene expression profiles of D39 pneumococci and human PMC during initial interactions *in vitro*. The application of stringent filtering criteria identified up- and down-regulated expression of particular sets of pneumococcal genes associated with bacterial fitness/metabolism and adherence mechanisms. For example, up-regulation of genes in the Spn *glnR* regulon was observed: glutamine metabolism has been reported to play a role in streptococcal virulence by providing the cell with a source of nitrogen [20] and pneumococci deficient in *glnA-glnP* were attenuated in their ability to colonise the murine nasopharynx and showed reduced adherence to Detroit 562 human pharyngeal epithelial cells [14,20]. In addition, the product of *glnQ* has been shown to mediate adherence of group B streptococci to host cells by binding to fibronectin [21]. A role for the *aliA* gene product in cell adhesion has been reported and mutations within the *aliA* gene decreased pneumococcal binding to pulmonary epithelial cells via the GalNAc-β-1-4Gal glycoconjugate receptor [22]. Since the pneumococcus has to adapt to the varying availability of micronutrients at discrete host niches, including the pleurae, it is possible that virulence gene expression is modulated by metabolic regulators. Our observation of increased *psaB* gene expression is similar to Orihuela *et al’s*. observation of increased *psaB* expression following bacterial association with pharyngeal epithelial cells [17]. In addition, Song *et al*. reported the induction of *psaC* following colonization of lung epithelial cells [23]. Although we found no change in *psaA* gene expression in our study, a requirement for proteins of the *psa* operon for infection of the mouse lung has been shown with mutants lacking *psaA*, *psaB* and *psaC* expression [24].

The expression of other genes that may play roles in promoting adherence, e.g. *lytB* and *nox* was up-regulated in Spn. In our study, down-regulation of CPS subunit genes *cpsH*, *I*, *J*, *L* and *O* (in two out of three experiments), may potentially favor pneumococcal adhesion [25]. Conversely, we observed up-regulation in *ugd* gene expression, encoding a UDP-glucose dehydrogenase essential for CPS biosynthesis and it is possible that gene expression for virulence factors is not steady-state within a population of D39 bacteria in various stages of interaction with human cells. The *lytB* gene encodes for a murein hydrolase that has been reported to be important for pneumococcal attachment to human nasopharyngeal cells [26] and rat nasopharynges. NADH oxidase, the product of the *nox* gene, has been reported to be a putative adhesin for pneumococcal adherence to A549 epithelial cells [27] and a *nox* mutant was attenuated for virulence in a mouse model of streptococcal respiratory tract infection [28].

Several genes associated with metabolic processes and bacterial fitness were up-regulated in Spn. Up-regulation of the adenylate kinase encoding gene *adK* has been reported to be essential for D39 growth [29] and up-regulation of the F0F1 ATPase related genes *atpB*, *atpF* and *atpH* may be important for streptococci maintaining intracellular pH and membrane potential [30]. Up-regulation of the *czcD* gene has been reported to occur under conditions of Mn2+ stress and the importance of Mn^2+^ to pneumococcal virulence is becoming increasingly apparent, particularly its role in the regulation of the oxidative-stress-response genes *spxB* and *sod* (superoxide dismutase) [31,32]. Furthermore, several genes encoding hypothetical products of unknown function and ABC-transporter proteins were also up-regulated in D39 following initial interactions and mutants for many of these genes, e.g. *sp_0145*, *sp_1856*, *sp_1715*, *sp_1855* (*adhB*), *sp_1538* (PPIase) and *ilvB*, were identified using a signature-tagged mutagenesis approach as attenuated for infection of a murine model of streptococcal pneumonia [33].

Down-regulation was observed also for Spn genes associated with metabolic processes and stress. Expression of *adh* (alcohol dehydrogenase) related genes was down-regulated in D39 interacting with PMC and a recent study has shown that D39 virulence was attenuated in *adhE* mutant bacteria, which correlated with host cell survival by reducing myeloperoxidase activity, inflammatory cytokine secretion and inflammation [34]. We observed down-regulation of *trpD*, *sp_1859*, *lacA*, *sp_1988* and *purL* gene expression and mutations in these genes attenuated streptococcal infection of murine models [33,35]. Differential regulation of purine ribonucleotide biosynthesis genes was highlighted by Song *et al*. [23], who observed down-regulation of 11 pneumococcal genes involved in this process in the avirulent strain R6 following interaction with human lung cells. The *ccdA-trxA* genes are believed to be involved in mediating resistance to stress [36] and it is possible that down-regulated expression of the gene encoding for a glycerol uptake protein may be host-protective against Spn-induced cytotoxicity. Furthermore, attenuation of the activity of an associated glucose metabolism enzyme, glpD, may be host-protective by reducing the formation of cytotoxic H_2_O_2_ [37]. We also observed down-regulation of *arcA* gene (in 2/3 experiments) encoding for arginine deiminase, which is part of the arcABCDT arginine deiminase system: D39 deficient in *arcD* gene expression led to increased adherence of bacteria to A549 epithelial cells [38]. Down-regulation was observed for genes encoding hyaluronidase (*hysA*) and GAPDH (*gapN*); down-regulation of the former may reduce pneumolysin cytotoxicity [39] and of the latter, the likelihood of endowing D39 with proteolytic activity [40], thereby favoring host cell survival.

During initial PMC infection, no differential expression was shown for other virulence-associated D39 genes such as *blpU* (*sp_tigr4-0041*, bacteriocin), *blpK* (*sptigr4-0533*, bacteriocin associated protein), *pspA* (*sp_tigr4-0117*, Spn surface protein A), choline-binding proteins (*cbpA*, *sp_tigr_2190*; *cbpD*, *sptigr4-2201*; *cbpJ*, *sp_tigr4-0378*; *cbpG*, *sp_tigr4-0390*; *cbpF*, *sp_tigr4-0391*), LPXTG-anchored *prtA* (*sp_tigr4-0641*,Protective antigen A), *xseA* (*sp_tigr4-1207*, Exodeoxyribonuclease VII, large subunit), *htrA* (*sp_tigr4-2239*, Serine protease), *spoJ* (*sp_tigr4-2240*, sporulation protein homologue), *spxB* (*sp_tigr4-0730*, encoding a pyruvate oxidase) [41], neuraminidase, the PPIases SlrA and PpmA, lipoproteins Pia and Piu, and glycolytic enzymes and enolase [42,43]. Our findings agree with the observations from other studies that patterns of expression for virulence factor genes and gene products differ between pneumococci isolated from distinct human and animal host niches [17,43,44] and vary also between different pneumococcal serotypes [45].

Early D39 interactions trigger changes in expression for a large number of PMC human genes within canonical pathways (S3A and S3B Table) and IPA analysis allows the specific genes that show changes in regulation to be grouped into network maps according to pathway/disease associations (S2 Fig). Beneficial to the host are likely to be regulation of expression of genes within canonical pathways associated with cellular survival stress responses [46]. We observed regulation of genes of the EIF-2 phosphorylation pathway and similar pathway regulation has been reported to contain damage to intestinal epithelia infected with *Clostridium difficile* [47] and defective EIF2 signaling led to more invasion of host cells by diverse organisms such as *Yersinia*, *Listeria monocytogenes* and *Chlamydia trachomatis* [48]. Regulation of genes associated with host cell mitochondrial function, via the oxidative phosphorylation, stress and mitochondrial dysfunction pathways, was also observed in response to D39. Similar regulation of an oxidative phosphorylation pathway was reported during early infection of the intestinal mucosa of mice with *Salmonella typhimurium* [49] and *P*. *aeruginosa* infection of *Caenorhabditis elegans* caused mitochondrial dysfunction that eventually led to induction of innate immune responses [46]. Regulation of genes for mRNA binding translation factors (eIF4) and p70S6K signaling pathways may be involved in influencing cellular growth, since over- and under-expression of eIF4 has been reported to lead to disordered and reduced rates of cellular growth, respectively [50]. Expression of genes associated with mTOR (mammalian target of rapamycin) was also observed in PMC cells in response to D39: mTOR is a master regulator that transmits signals that inhibit autophagy [51], which has been reported to play a role in innate immune responses to intracellular pathogens such as *S*.*typhimurium* [52]. Up-regulation of genes in the ubiquitination pathway suggests also a host cell defense mechanism [53]. By contrast, regulation of genes involved in remodeling of epithelial adherens junctions may provide a mechanism whereby D39 traverses cellular barriers in the absence of significant intracellular invasion [54].

In addition to the PMC canonical pathways, the top 10 up- and down-regulated genes were also examined. Although these represent only a minor subset of the global gene expression changes and were chosen to validate the microarray analyses, the biological significance of these genes appears to suggest that the cellular response is directed towards host defense and survival. D39 interaction up-regulated expression in PMC for the gene encoding the diazepam binding inhibitor protein, which has a role in increasing host cell numbers and increased expression of the *CNOT6* and *ATF4 g*enes has been reported to be important for protecting cells from infection stress and death [55,56]. Up-regulated expression of connective tissue growth factor *CTFG* was also observed in our study and has been suggested as a common host response to bacterial contact, enabling host cells to adapt to the stress of infection [57]. Increased expression of genes encoding ribosomal proteins, transcriptional factors and the chaperone Hsp40 protein are also important cellular stress responses. Up-regulation of the *OPTN* gene that functions in maintenance of the Golgi complex was observed in D39-infected PMC and Wild *et al* reported that OPTN protein can function as an autophagic receptor, for example to restrict the growth of *Salmonella* [58]. D39 interaction also up-regulated expression of the *NCL* gene in PMC, encoding nucleolin, an abundant acidic phosphoprotein found in exponentially growing cells: interestingly, expression of nucleolin has been reported to promote *Fransicella tularensis* infection of monocytes [59], but whether this occurs during pneumococcal infection remains to be determined.

D39 interactions led to down-regulated expression of apoptosis-related genes, e.g. *SET* [60], *HNRNPC* [61], *OAZ1* [62] and *SNRPB*, which potentially favors cell survival. Down-regulated expression of the gene encoding the translation regulator EEF1A1 may also be important for cell survival as shown by the example of *Legionella pneumophila* glucosylation of EEF1A1 in mammalian cells *in vitro* by the Lgt1 glucosyltransferase protein, which blocks protein synthesis and induces cell death [63]. Down-regulating expression of the *GPX4* gene may be important for host cellular protection against oxidative stress, since stress can be transduced by glutathione peroxidase into a cell-death pathway [64]. D39 infection down-regulated expression of the S phase-specific cell cycle thymidine kinase 1 (*TK1*) gene, which may be related both to cell senescence [65] and a consequence of uncoupled regulation by HNRNPC [66]. Down-regulated expression was also observed for PMC genes encoding tetherin (*BST2*), β-actin (*ACTB*) and the ADP-ribosylating factor (*ARF4*). Although the role of *BST2* in bacterial infections is unclear, BST2 gene product has been reported as an antiviral factor whose expression inhibits virus release from infected cells [67]. It is generally accepted that many bacterial pathogens use the β-actin cytoskeleton to promote intimate pathogen attachment to the host cell plasma membrane and cellular invasion [68]; in addition, down-regulated *ARF4* gene expression in HeLa cells resulted in defects in persistence and growth of the intracellular pathogens *C*.*trachomatis* and *Shigella flexneri* [69].

Empyema appears to be an inflammatory condition caused by planktonic pneumococci and immune cells that interact with PMC and infiltrate into the pleural cavity. In the current study, infection of PMC with live pneumococci did not induce an innate immune response *in vitro*, as demonstrated by the absence of inflammatory cytokine gene and protein expression and the lack of NF-kB or p38 MAPK activation. Wilkosz and colleagues also did not report significant cytokine production by Met-5A cells *in vitro* following D39 infection [6]. This lack of innate response to pneumococcal infection has been observed with other human cells infected *in vitro* with pneumococci, including human meningeal, pulmonary epithelial [12,70] and peripheral blood mononuclear [71] cells. However, PMC can respond to exogenous stimuli such as LPS-replete Nm-OM or TNFα, both of which activate NF-kB and p38 MAPK, and secrete cytokines. We did observe cytokine production with heat-inactivated D39, but this response required the administration of a high MOI-equivalent of bacteria that is unlikely to be reached during infection with live bacteria and hence biologically irrelevant. Significantly, live pneumococcal infection abrogated cytokine production induced by exogenous stimuli and coupled with the lack of cytokine induction by treatment with a D39 bacterial lysate and a sterile filtrate, suggests that a heat-modifiable molecule could be involved in supressing innate responses. Although the inhibitory mechanism is not known, pneumolysin treatment of human epithelial cells for example, selectively regulated the expression of MAPK phosphatase 1, which inhibited the p38 MAPK signalling pathway by inactivating p38 MAPK by dephosphorylation [72]. The absence of cytokine production in the PMC model, but the presence of cytokines in the pleural fluid of empyema patients and infected animals [6], suggests that sentinel cells possibly within the pleural fluid itself function in innate recognition of invading pneumococci and initiate an inflammatory response, which is subsequently exacerbated by infiltrating immune effector cells.

The current study presents several caveats: *in vitro* patterns of microarray gene expression may not mimic the patterns observed *in vivo*, although it is likely to be difficult to isolate pneumococci in sufficient numbers from infected pleural fluid taken from patients with invasive disease, to provide bacterial RNA for analyses. Furthermore, RT-qPCR validation of gene expression was not always consistent, which has been reported elsewhere [73] and emphasizes the importance of ultimately demonstrating biologically functional roles for the expressed proteins during empyema. In our study, correlation between RT-qPCR and microarray data was observed for up- and down-regulated expression of 12 out of 24 pneumococcal genes and 16 out of 20 human genes. In some cases, there were significant differences in the fold variations between RT-qPCR and microarray analysis, *e*.*g*. for pneumococcal genes *sp_0858*, *sp_1914*, *sp_1178*, *sp_1775*, *trxA and trpD*. Factors that may influence the correlation between these two methods of measuring gene expression include the use of different normalization procedures and data quality parameters [73]. Using untransformed PMC perhaps would confirm the findings in this study and also the study from Wilkosz and colleagues [6], but isolating primary paediatric or adult PMC in sufficient quantities to establish reproducible *in vitro* models would be a considerable challenge. Regardless, our *in vitro* model did enable gene expression to be compared between adherent pneumococci and control bacteria and between infected and control PMC cells, whereas similar controls for clinical disease samples would be difficult to obtain. It should be stressed that a lack of differential expression does not eliminate the possibility that putative virulence genes are involved in pleural cell interaction and in the pathogenesis of empyema. As an alternative to microarrays, RNA-sequencing could be used to study genome-wide analysis of differential gene expression in PMC and D39: this may offer the advantage of better sensitivity for genes with low expression, although gene expression–based predictive models generated from RNA-sequencing and microarray data are essentially similar [74].

In summary, the current pilot and descriptive study provides datasets and a platform for examining further the molecular mechanisms underlying the pathogenesis of empyema.

## Supporting Information

S1 Fig Immunohistochemistry for cell markers specific to Met-5A cells. (PPTX)

S2 Fig Ingenuity Pathway Analysis of Met-5A gene networks defined by top disease and functions with corresponding Gene Network Maps. (PDF)

S1 Table Nucleotide sequences for primers used for RT-qPCR of selected *Streptococcus pneumoniae* D39 and human Met-5A genes. (DOCX)

S2 Table S2A Table. Full gene expression lists for *Streptococcus pneumoniae* D39 for each microarray experiment. S2B Table. *Streptococcus pneumoniae* D39 genes showing > 2-fold changes in each experiment after normalization. (XLSX)

S3 Table S3A Table. List of average gene changes in human PMC following infection with *Streptococcus pneumoniae* D39. S3B Table. Canonical Pathways identified by Ingenuity showing numbers of up- and down-regulated genes from n = 5 independent experiments. (XLSX)
