# Supplementary material for: Co-Transcriptomes of Initial Interactions In Vitro between Streptococcus Pneumoniae and Human Pleural Mesothelial Cells
Source: PLoS One. 2015 Nov 13;10(11):e0142773. doi: 10.1371/journal.pone.0142773 (PMC4643877; doi:10.1371/journal.pone.0142773)
Supplement: S1 Fig — (PPTX) [file pone.0142773.s001.pptx]

## Slide 1
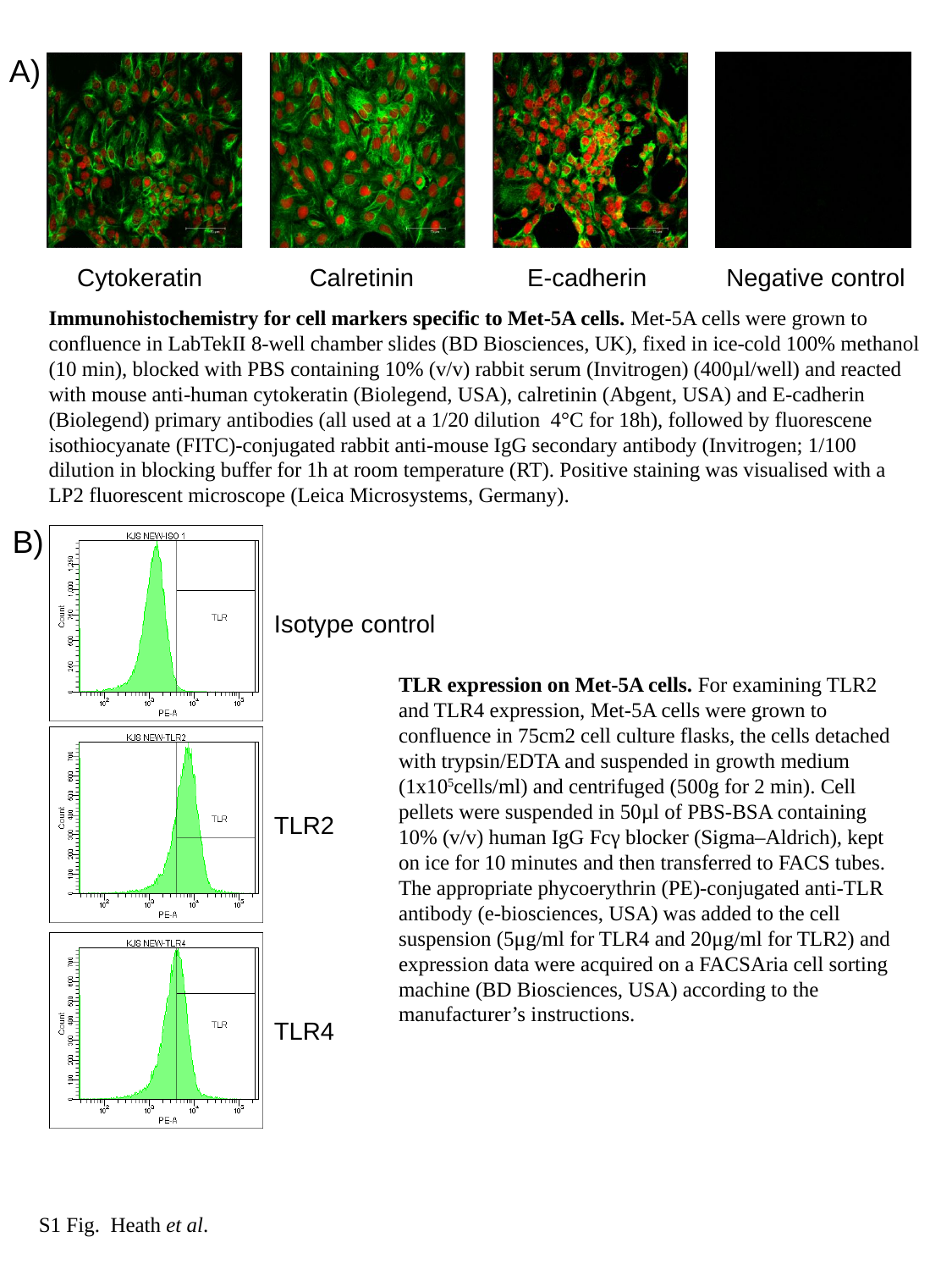

A)
Cytokeratin
Calretinin
E-cadherin
Negative control
Immunohistochemistry for cell markers specific to Met-5A cells. Met-5A cells were grown to confluence in LabTekII 8-well chamber slides (BD Biosciences, UK), fixed in ice-cold 100% methanol (10 min), blocked with PBS containing 10% (v/v) rabbit serum (Invitrogen) (400µl/well) and reacted with mouse anti-human cytokeratin (Biolegend, USA), calretinin (Abgent, USA) and E-cadherin (Biolegend) primary antibodies (all used at a 1/20 dilution 4°C for 18h), followed by fluorescene isothiocyanate (FITC)-conjugated rabbit anti-mouse IgG secondary antibody (Invitrogen; 1/100 dilution in blocking buffer for 1h at room temperature (RT). Positive staining was visualised with a LP2 fluorescent microscope (Leica Microsystems, Germany).
B)
Isotype control
TLR2
TLR4
TLR expression on Met-5A cells. For examining TLR2 and TLR4 expression, Met-5A cells were grown to confluence in 75cm2 cell culture flasks, the cells detached with trypsin/EDTA and suspended in growth medium (1x10^5^cells/ml) and centrifuged (500g for 2 min). Cell pellets were suspended in 50µl of PBS-BSA containing 10% (v/v) human IgG Fcγ blocker (Sigma–Aldrich), kept on ice for 10 minutes and then transferred to FACS tubes. The appropriate phycoerythrin (PE)-conjugated anti-TLR antibody (e-biosciences, USA) was added to the cell suspension (5μg/ml for TLR4 and 20μg/ml for TLR2) and expression data were acquired on a FACSAria cell sorting machine (BD Biosciences, USA) according to the manufacturer’s instructions.
S1 Fig. Heath et al.
