# Supplementary material for: Co-Transcriptomes of Initial Interactions In Vitro between Streptococcus Pneumoniae and Human Pleural Mesothelial Cells
Source: PLoS One. 2015 Nov 13;10(11):e0142773. doi: 10.1371/journal.pone.0142773 (PMC4643877; doi:10.1371/journal.pone.0142773)

Top Diseases and Functions and Network Map	Molecules in Network	Score	Focus Molecules
Developmental Disorder, Hereditary Disorder, Metabolic Disease<br>Network Map 1	Akt,ASXL1,ASXL2,CCNG1,COPS3,COPS6,CUL3,ETFA,GNRH2,HN1, Mitochondrial complex 1,NADH dehydrogenase,NAE1,NDUFA3,NDUFA6, NDUFA7,NDUFB1,NDUFB2,NDUFB3,NDUFB4,NDUFB8,NDUFS5,NDUFS6, NDUFV1,NDUFV2,NEDD8,PBK,PHF1,RNF7,RPL11,RPLP0,RPLP1,RPLP2,STOML2,WDR48	42	32
Gene Expression, Cellular Development, Tissue Development<br>Network Map 2	ATF4,Ck2,Cytoplasmic Dynein,DYNLL1,DYNLRB1,DYNLT1,HMGN1, HPRT1,HTATSF1,IHH,MED22,mediator,NAP1L1,NPM1,PABPN1,POLR2B,POLR2F,POLR2G,POLR2I,POLR2K,POLR2L,RAD51AP1,RBX1,RNA polymerase II,RPL12,RPS21,SDHC,SLC38A2,SSRP1,TAP2,TBC1D8,TCEB2,TPI1,Vegf,ZNF268	38	30
RNA Post-Transcriptional Modification, Molecular Transport, RNA Trafficking<br>Network Map 3	ALK,CD59,CDC42SE1,CYR61,DDX39A,EIF4A,EIF4A2,EIF4A3,EIF4F,EIF4G2,ERH,Ferritin,FTH1,FTL,H2AFY,hemoglobin,ILF3,Jnk,LOC102724594/U2AF1,PAIP1,PCBP2,RBM8A,RPL4,SAP18,SF3B1,SRSF1,SRSF3,SRSF4,SRSF7,SRSF9,THOC3,TRA2B,TSH,VEGFB,ZAK	36	29
Free Radical Scavenging, Small Molecule Biochemistry, Cell Morphology<br>Network Map 4	Aldose Reductase,ATP6AP2,C1QBP,C4orf27,CAMK2G,CD99,COX4I1, COX5A,COX6A1,COX7A2,COX7C,COX8A,Cytochrome bc1,cytochrome-c oxidase,FAM103A1,FLII,GAPDH,glutathione peroxidase,GPX4,GST,HDGF,Ldh (complex),MGST1,MGST3,NFkB (complex),NGFRAP1,PARK7, PLP2,PRDX3,PRDX6,RTKN,SEPT9,STK10,VMP1,ZMYND11	34	28
Organismal Injury and Abnormalities, Cellular Compromise, Inflammatory Disease<br>Network Map 5	ACSL3,APP,ATP5H,Creb,DERL2,ECH1,H3F3A/H3F3B,Histone h3,HOXD4,IL12(complex),Immunoglobulin,LMNA,MICAL2,NARF,NAV1,NCL,NFE2L2,Nr1h,PDHA1,PPIB,RPL7,RPL18,RPS11,SLC11A1,SOD1,Sos,SPATS2L,TCF,THUMPD3,UQCRQ,VCP,WDR1,WDR33,WRB,YY1	34	28
Protein Synthesis, Gene Expression, Cellular Assembly and Organization<br>Network Map 6	Actin,ACTR3,ADAM33,Alphacatenin,Arp2/3,ARPC2,ARPC5,ARPC1A,Cadherin,CDH15,CKS2,CLIC1,CTNNA1,CTNND1,EEF1A1,EEF1B2,EEF1G,ERK,FAU,Igfbp,IGFBP7,LAMA4,MAGED1,MKNK2,PDGF(family),PTRH2,RhoGap,Rnr, RPL8,RPS16,RPS20,RPS28,RPS29,RPS3A,SLC20A2	30	26
Cell Cycle, Cellular Assembly and Organization, DNA Replication, Recombination, and Repair<br>Network Map 7	APC(complex),AURKA,BUB1,BUB3,CCNB2,Cdk,CDK1,CKAP2,CKS1B,Cyclin A,Cyclin B,Cyclin E,GABPB1,Histone H1,HNRNPM,Importin beta,IPO5, MAD2L2,MORF4L2,MYBL2,PELP1,PHB2,PI3K(complex),PTMA,RNA polymeraseI,RPA2,RPL13,RPL23,RPL27,RPL18A,SKP2,SLC12A4,SNRPD3,SUMO2,TPX2	30	26
Gene Expression, Protein Synthesis, Developmental Disorder<br>Network Map 8	19S proteasome,20s proteasome,26s Proteasome,AHSA1,ATF6B, BST2,CACYBP,EIF1,EIF3,EIF3E,EIF3H,EIF3K,EIF3L,EIF3M,ERK1/2,KARS, MARS,MHC CLASS I (family),MPZL1,POMP,PSMA,PSMA2, PSMA4,PSMB,PSMB2,PSMB5,PSMB6,PSMC,PSMC1,PSMC2,PSMC6,PSMD8,PSMD9,TK1,Ubiquitin	28	25
Cellular Function and Maintenance, Cancer, Respiratory Disease<br>Network Map 9	aldo,ALDOA,ALG5,Ap2alpha,AP2S1,ATP6V0E1,ATP6V1E1,ATP6V1F,CDC45,CDK4,CDKN2C,CENPK,CyclinD,DONSON,Dynamin,E2f,E2F2,Mapk,MARCKS,MDK,OTUB1,PCNA,PIP5K1C,POLE,Rb,RFC4,RPA,RPSA,SERF2,SERINC3,TMEM126A,UBE2,UBE2C,UBE2I,Vacuolar H+ ATPase	28	25
Cell-To-Cell Signalling and Interaction, Reproductive System Development and Function, Cellular Assembly and Organization<br>Network Map 10	14-3-3,ACTB,Alpha tubulin,Arf,ARF4,CAPZB,Caveolin,CCT2,CCT4,CCT5, CCT7,CCT8,CD9,CELA2A,CST3,CSTB,DCTN2,DSN1,Dynein,elastase,ENaC,FNTA,GABARAP,GNAI2,MVP,Pak,PLXNB1,RAC1,RGS12,TUBA1A,TUBA1B, TUBA3C/TUBA3D,tubulin (complex),tubulin (family),ZWINT	28	25

S2 Fig. IPA analysis of Met-5A gene networks defined by top disease and functions with corresponding Gene Network Maps shown below.

Network Map 1

Developmental Disorder, Hereditary Disorder, Metabolic Disease

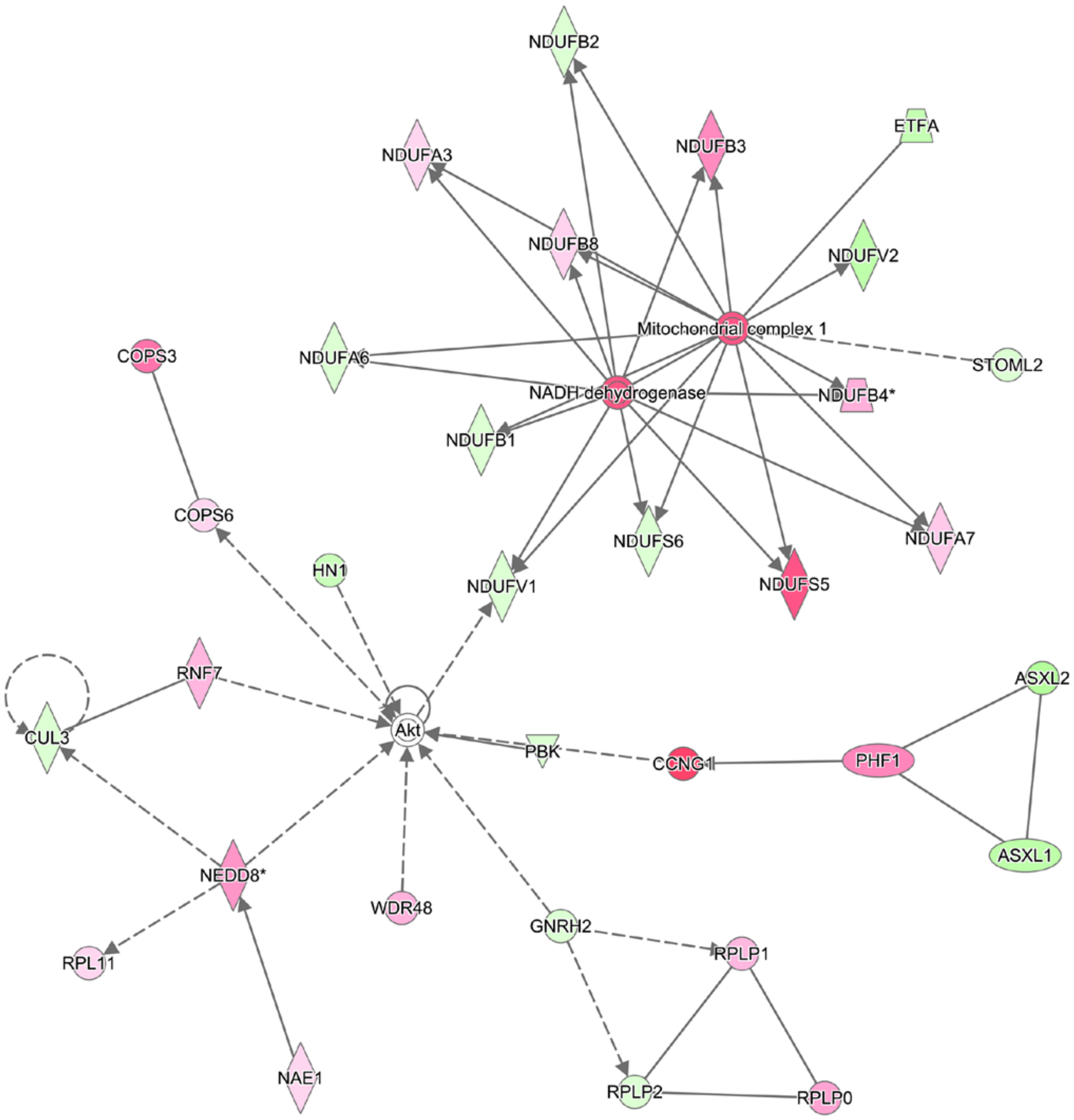

Network Map 2

Gene Expression, Cellular Development, Tissue Development

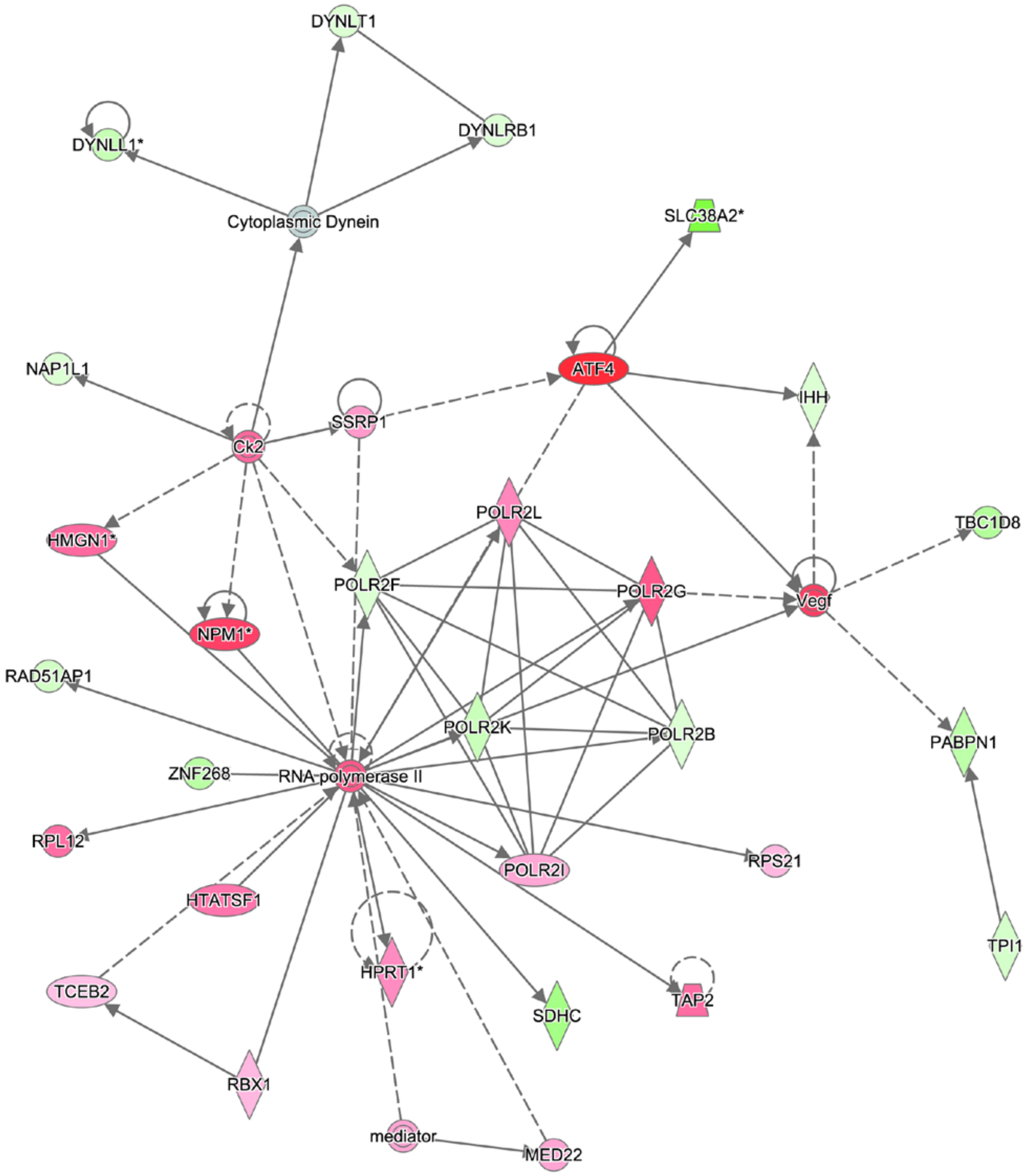

Network Map 3

RNA Post-Transcriptional Modification, Molecular Transport, RNA Trafficking

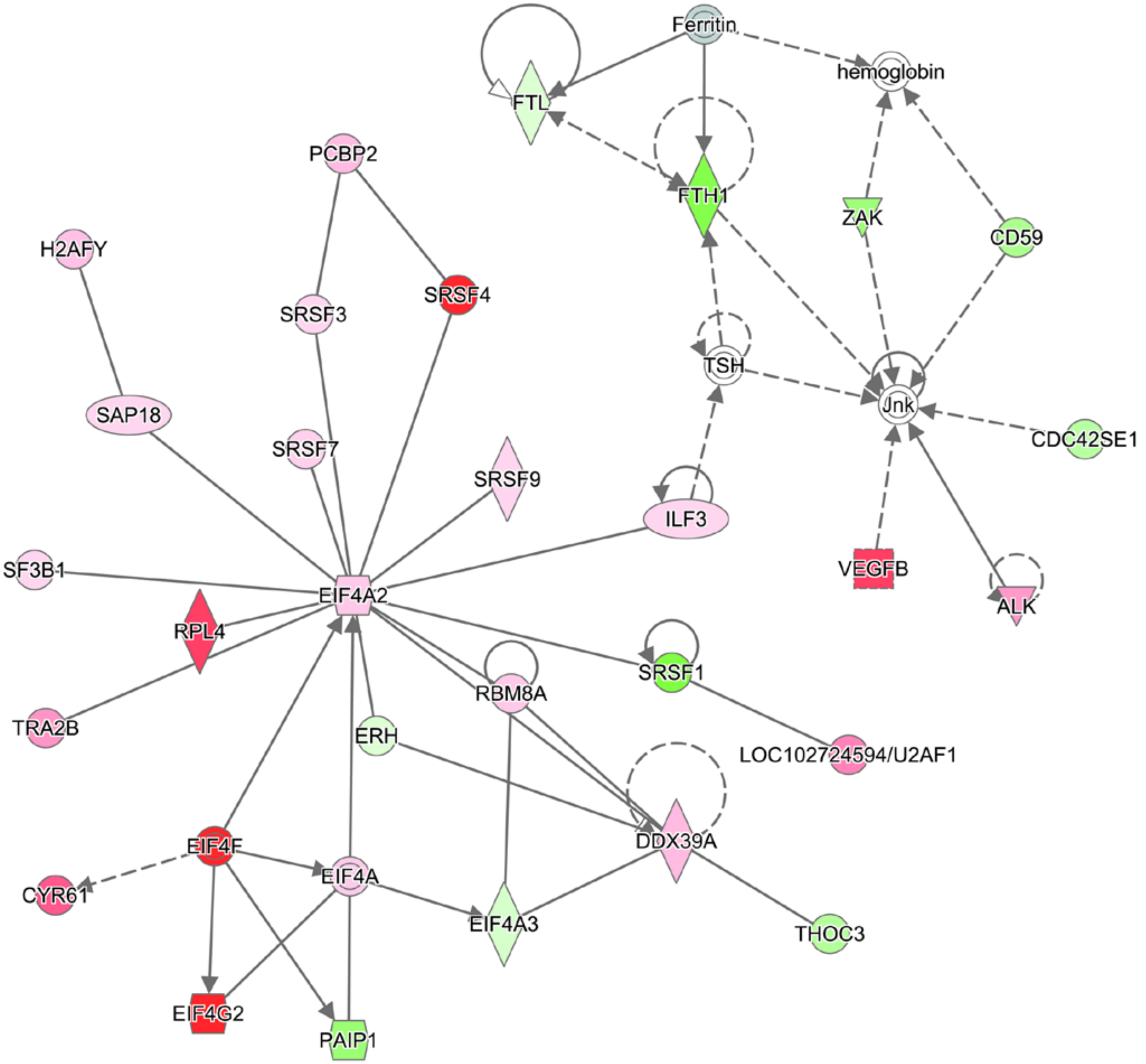

Network Map 4

Free Radical Scavenging, Small Molecule Biochemistry, Cell Morphology

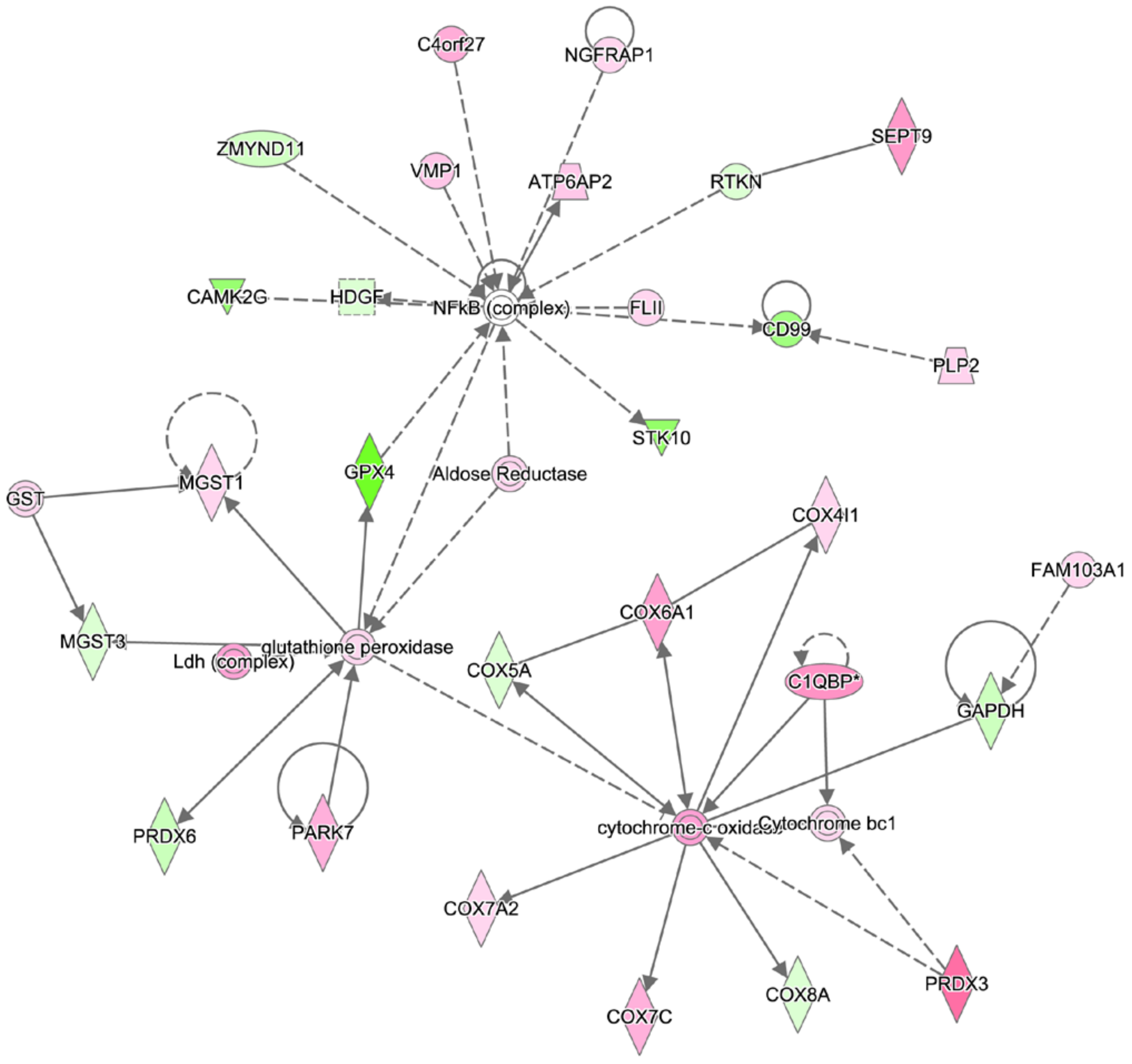

Network Map 5

Organismal Injury and Abnormalities,
Cellular Compromise, Inflammatory Disease

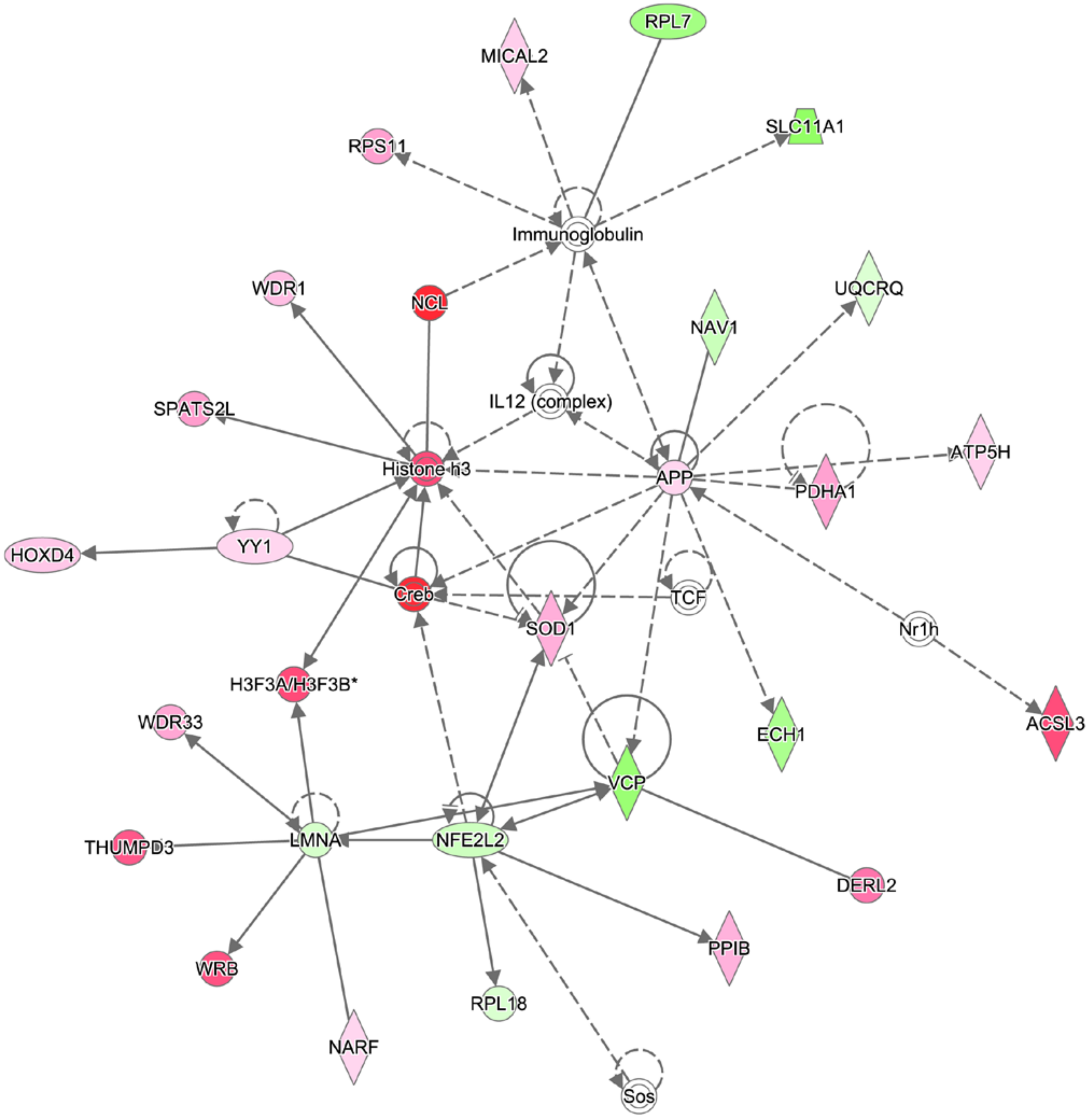

Network Map 6

Protein Synthesis, Gene Expression, Cellular Assembly and Organization

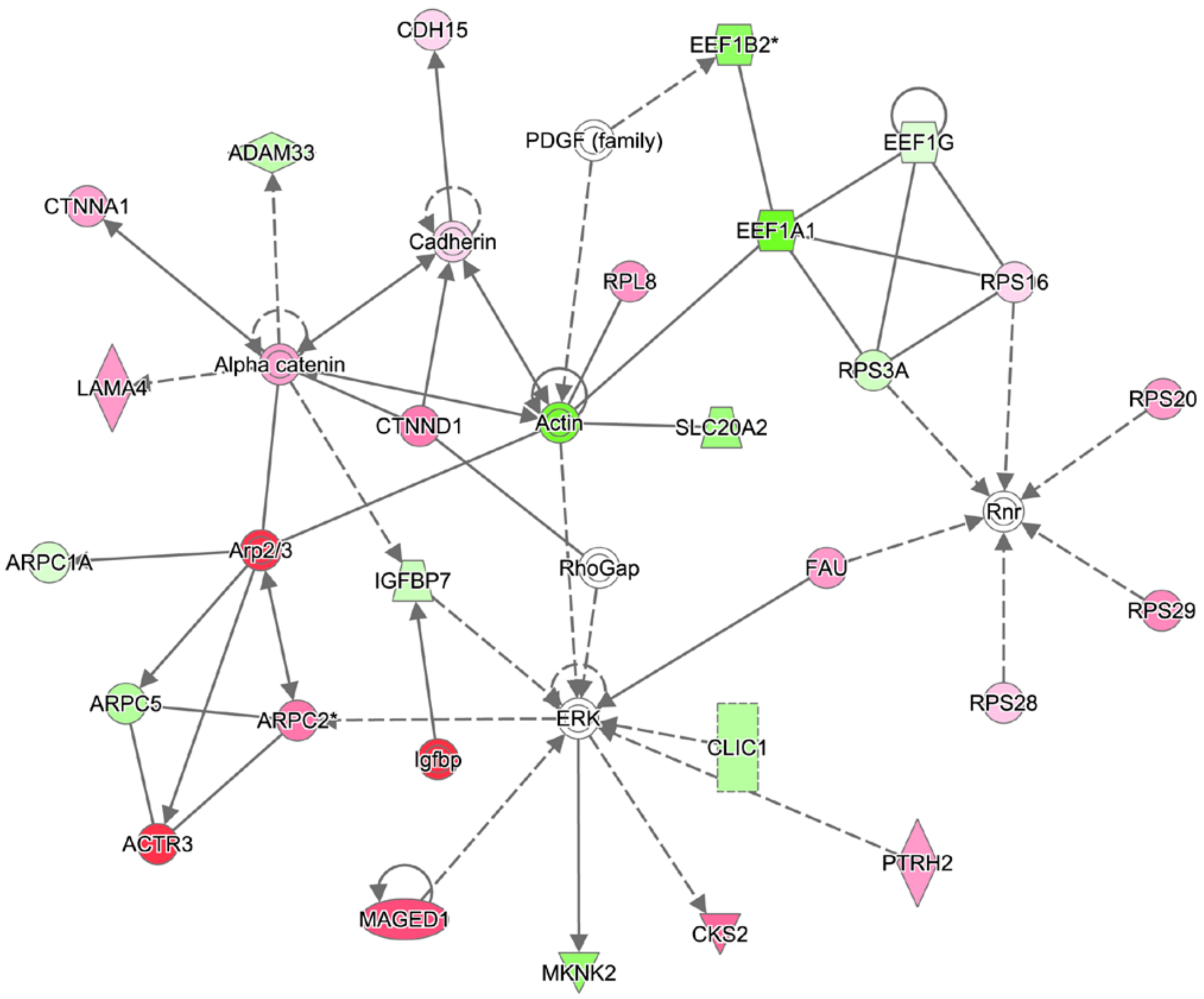

Network Map 7

Cell Cycle, Cellular Assembly and Organization, DNA Replication, Recombination, and Repair

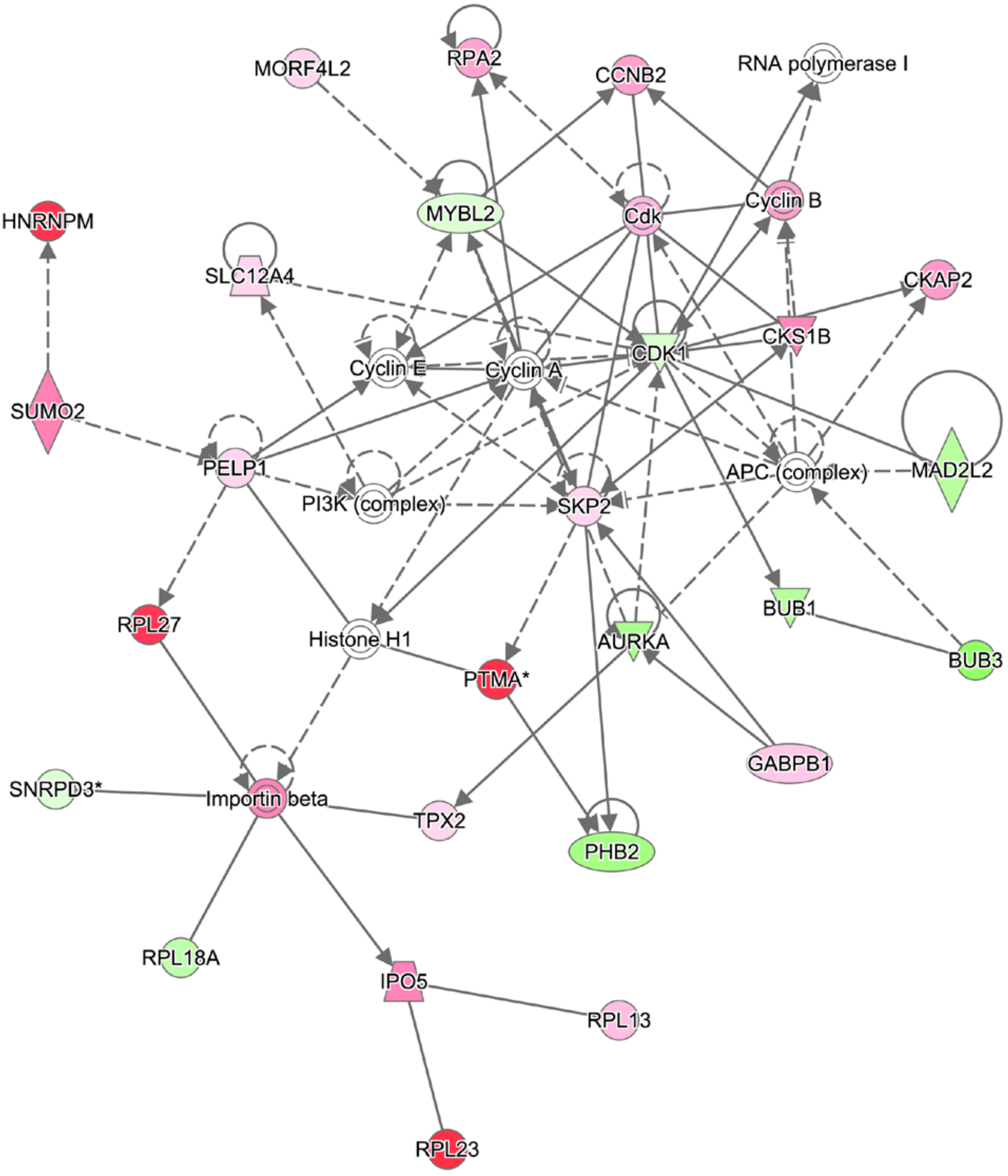

Network Map 8

Gene Expression, Protein Synthesis, Developmental Disorder

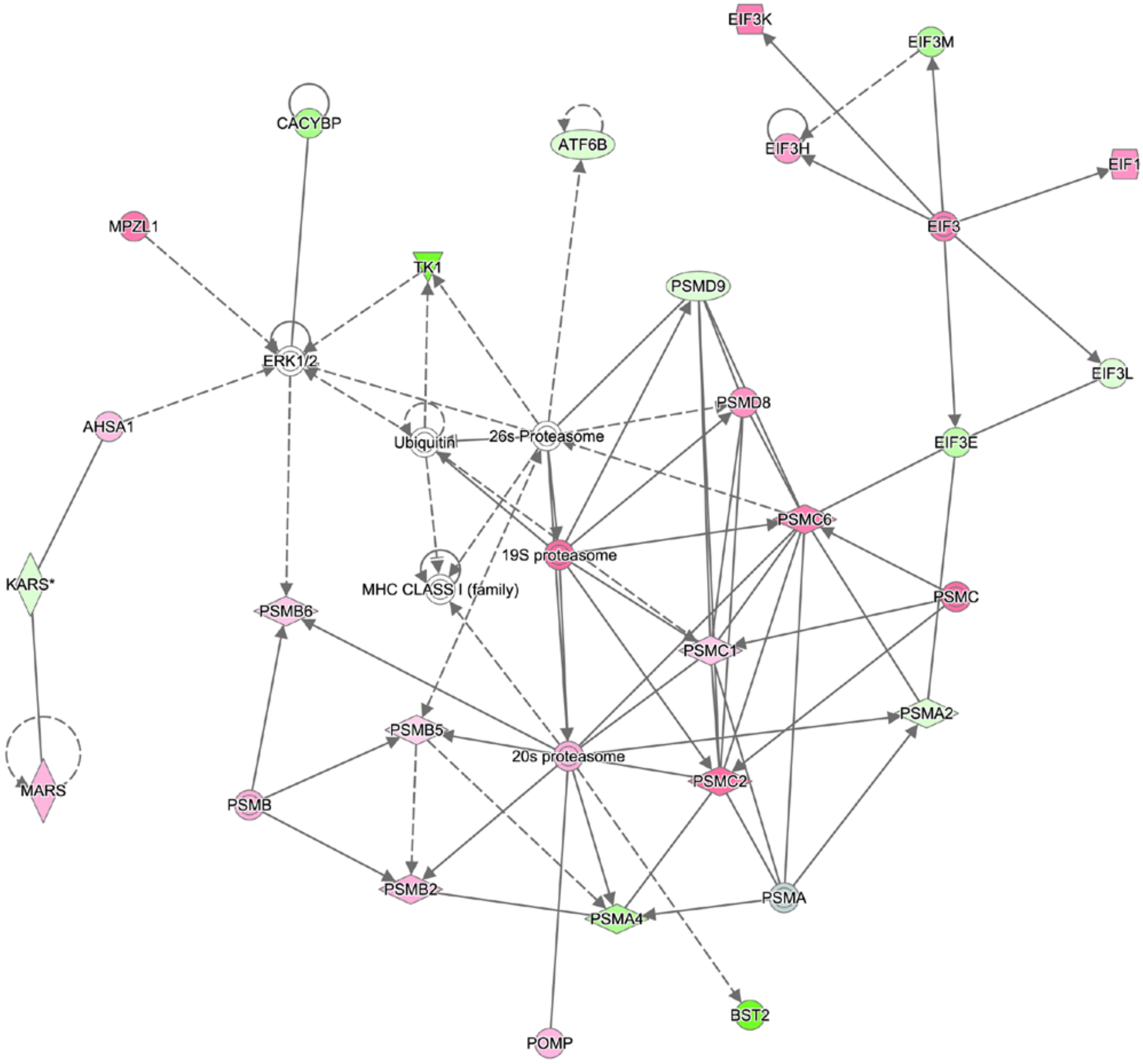

Network Map 9

Cellular Function and Maintenance, Cancer, Respiratory Disease

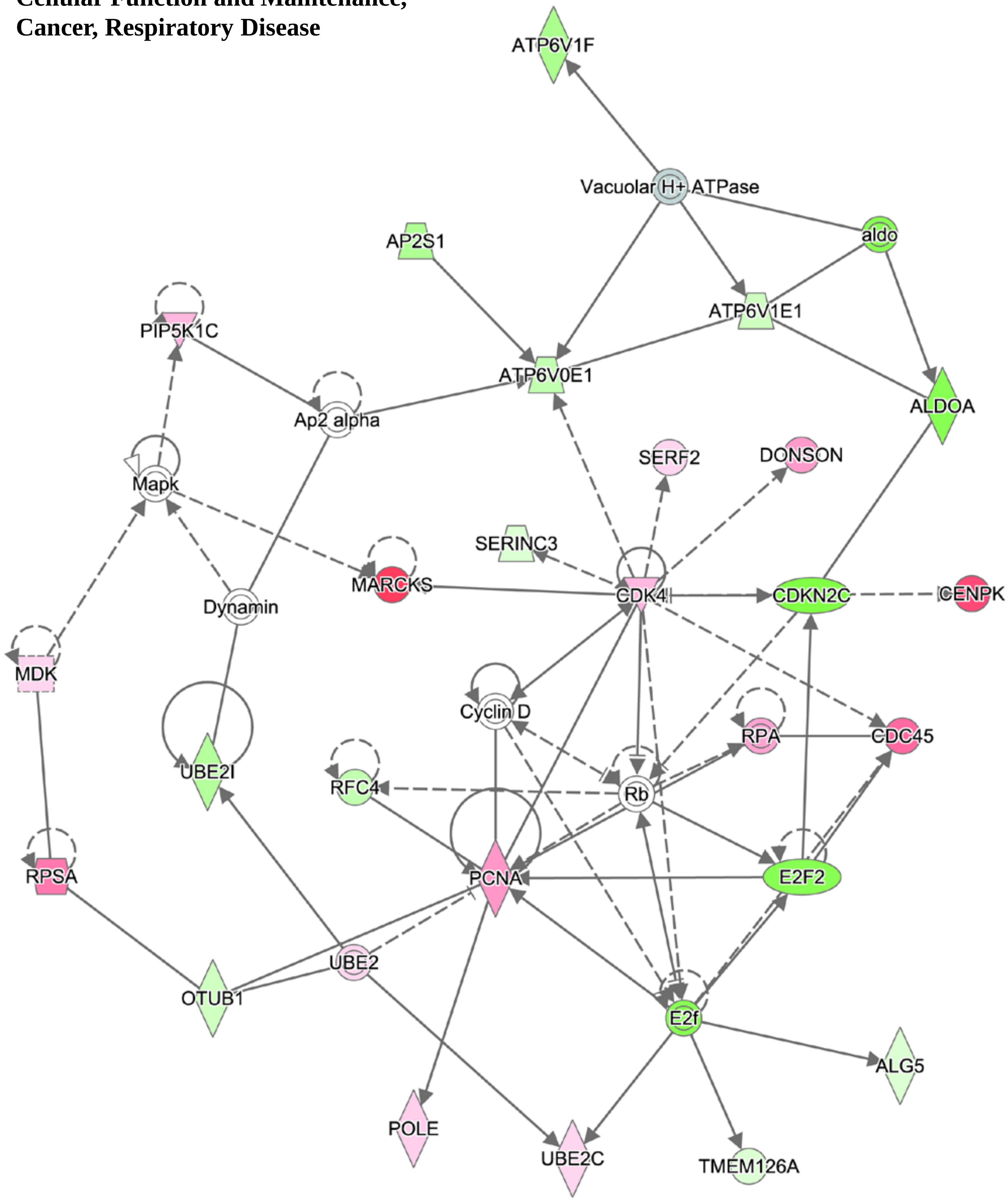

Network Map 10

Cell-To-Cell Signalling and Interaction, Reproductive System Development and Function, Cellular Assembly and Organization

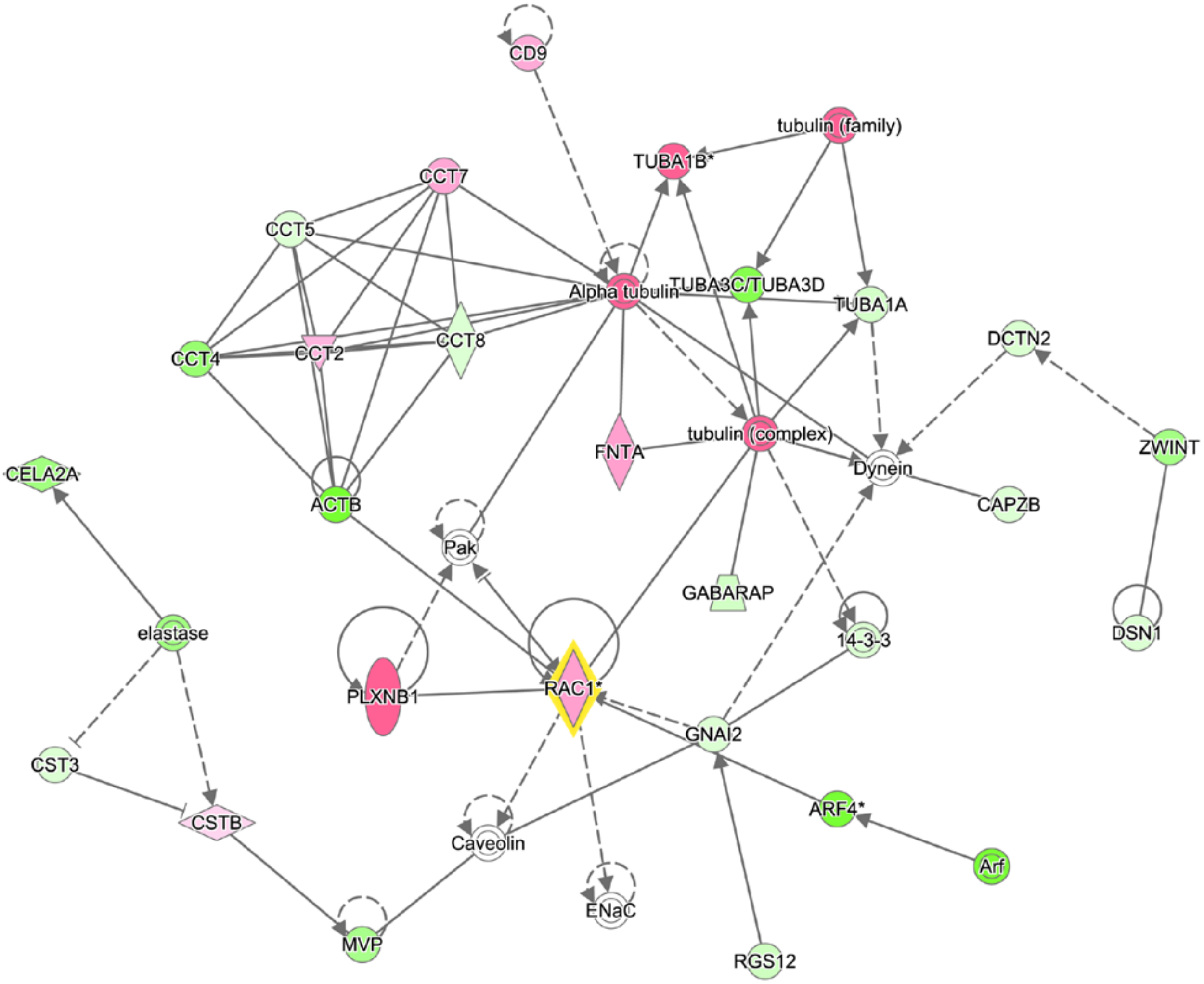

Supplement: S2 Fig — (PDF) [file pone.0142773.s002.pdf]
