# Supplementary material for: Co-Transcriptomes of Initial Interactions In Vitro between Streptococcus Pneumoniae and Human Pleural Mesothelial Cells
Source: PLoS One. 2015 Nov 13;10(11):e0142773. doi: 10.1371/journal.pone.0142773 (PMC4643877; doi:10.1371/journal.pone.0142773)
Supplement: S1 Table — (DOCX) [file pone.0142773.s003.docx]

| **Pneumococcal**  **gene** | **Sense primer sequence** | **Anti-sense primer sequence** | |  |
| --- | --- | --- | --- | --- |
| *gdhA* | TGCTCAAGGCGTTATCTGTG | AGCTTTTGCAGGTCCGTAGA | |  |
| *SP_1914* | GGGAATGCAACCGTCTATGT | CTTCCCAAAAGCCACATCTC | |  |
| *SP_0136* | CGAGAAGAGGAGAAGACCGATT | ACAACACCGTCCTGATGTGA | |  |
| *psaB* | AGGAATGCGTCTCGTTAGGA | TAGTCAGCTAGGCCGACGAT | |  |
| *SP_0858* | ACCTTTGGACTCAAGCAACG | GCTGCTTCTCCCATAGGTTG | |  |
| *glnQ* | GACCCTGAGATGGTTGGAGA | CGAGGGTGTTGTGGGTTATC | |  |
| *SP_0857* | GGTTCTGCCATGAAACACCT | GATGAAGCTGTTGGCATCAA | |  |
| *SP_1992* | AGTGGCTCCTAATGCTGCTC | TTGTTGCCACCACTAGACCA | |  |
| *pdxK* | TATCAAGGGAGGCAATCGTC | CAAAGGTACAACCTGCACCA | |  |
| *nox* | CTGTTGGTGACTGTGCGACT | TGTAGGCACCAACGATACCA | |  |
| *SPR1178* | CACCGAAATTTCCTCAGCTC | GAGGCAATGGCCTGTTCTAA | |  |
| *pdx1* | CTGATGACCGTTTCCATGTG | CTGTCCCTGGTTCTCCTTTG | |  |
| *glnA* | TCTTGAGTTGCGTTCAGTGG | TTGGCACCACTAGCATTCAC | |  |
| *lytB* | TCAAAGCAGATGGACAGCAC | GACGTTCACAAGCTAACCTGTCT | |  |
| *SP_0138* | GGTTACCCTTTGAGCCAACA | ACCTACCCCAAGAGCTGGTT | |  |
| *adhE* | TGCTCCTGAAAACTGTGTGC | GTTCAGCCATACCACCGTCT | |  |
| *adhP* | TTCTTGGGCACGAAGGTATC | CAACCCCTTCCTTGATGCTA | |  |
| *trpD* | GATATTCGTGGTGGGAATGC | CAACCCCTTCCTTGATGCTA | |  |
| *trxA* | ACTTCTGGGCAACTTGGTGT | GTTTGACAACTTGGCCGTCT | |  |
| *SP_1775* | GCCAGCAAGAACGAAAGAAC | TCCATAAGTCCCATGGTAGTCC | |  |
| *SP_2187* | CTCTGGGCTCACATCAGTCA | CGGAACCTTTCTCAGCAAAC | |  |
| *SP_1069* | CATGGCCGCTATTACAGACC | TTCACATTCGGTGTCAGAGC | |  |
| *purL* | ACTCCCAGAAGAGCGTCAAA | CGTCCAACAGAGCAGTCAAA | |  |
| *purF* | TCTACTTTGCTCGCCCTGAT | TAGGGAAGAATTGGGCACAC | |  |
| *gyrA* | TACGCCATGAGTGTTATCGTAGCG | ACTATCTCCATGGAACC | |  |
| **Human gene** | | **Sense primer sequence** | **Anti-sense primer sequence** |  |
| *DBI* | | CTGGGACAGAGGCTGAGTTTGAG | GCCCACAGTTGCTTGTTTGTAGTGG | |
| *OPTN* | | CCCAAACCTGGACACGTTTACCC | CCTCAAATCTCCCTTTCATGGCTTG | |
| *RPL6* | | GAAACCCGAAGCCAAGAAGGTTGA | CAAGGACAGGGTTGCGGCTG | |
| *EIF4G2* | | CGGAGGAGGAAGTAGGGGTG | GCTTCGTGCAGGAATCCATTTCTG | |
| *SRSF4* | | GGGAAGATCCTGGAGGTGGATC | CGCTCACCACAAAGGTCTTTGCC | |
| *DNAJB6* | | GCGGAGGCATATGAAGTGCTGTC | GACTGTCAAAATGACTTCCACCTCC | |
| *NCL* | | GGTAGAAGAAGATAGTGAAGATGGAGG | CCTTCTTTGCTGAGGTTGCAGCAG | |
| *CNOT6* | | CTGGGCAGAGCTTGAAATAAGTGG | GTCTGAAGGAATTCGGGACAGGG | |
| *ATF4* | | CTGGAGGTGGCCAAGCACTTCA | CTTGCTGTTGTTGGAGGGACTGAC | |
| *CTGF* | | GGGCCTCTTCTGTGACTTCGG | GACTCTCCGCTGCGGTACAC | |
| *SET* | | CAGAAGAGGTCAGAATTGATCGCC | CTCCCCAAGCAGTGCAGACAC | |
| *BST2* | | GCTGGGGATAGGAATTCTGGTGC | CACTGCCCGAAGGCCGTCC | |
| *EEF1A1* | | CCATCTGATCTATAAATGCGGTGGC | CAGTTTATC CAAGACCCAGGCATAC | |
| *HNRNPC* | | GGAATCTCAACACTCTTGTGGTCAAG | AACATACTGAACGAAGGCAAAGCCC | |
| *OAZ1* | | CCATGCCGCTCCTAAGCCTG | AGGAGCACCACCGAGGCC | |
| *GPX4* | | GGGACGACTGGCGCTGTG | GTTGGTGACGATGCACACGAAG | |
| *TK1* | | CAGAGTTGATGAGACGCGTCCG | CCGGTCATGTGTGCAGAAGCTG | |
| *ACTB* | | GCACCAGGGCGTGATGGTG | CGATGCCGTGCTCGATGGG | |
| *SNRPB* | | GGATGAGGTGCATCCTGCAGG | GGCTTGATCTTTCTGAACTCATCACAG | |
| *ARF4* | | CTGCTGGCAAGACAACCATTCTG | CCACCAACATCCCATACTGTGAAAC | |
| *GAPDH* | | CCCATCACCATCTTCCAGGAGC | CCCAGCCTTCTCCATGGTGG | |

**S1 Table. Nucleotide sequences for primers used for RT-qPCR of selected *Streptococcus pneumoniae* D39 and human Met-5A genes.**
